# Missing Landmark Estimation Using Reverse Engineering: Challenges and Potential Solutions for the Study of Hominin Long Bones

**DOI:** 10.1002/ajpa.70334

**Published:** 2026-08-03

**Authors:** Lloyd A. Courtenay, Julia Aramendi

**Affiliations:** ^1^ CNRS, PACEA UMR5199 Université de Bordeaux Pessac France; ^2^ Department D'història I Història de L'art Universitat Rovira i Virgili Tarragona Spain; ^3^ Laboratoire de Paléontologie, Évolution, Paléoécosystèmes et Paléoprimatologie (PALEVOPRIM), UMR, 7262 CNRS & Université de Poitiers Poitiers France

**Keywords:** geometric morphometrics, metalearning, missing data, regression, thin plate spline, virtual reconstruction

## Abstract

Fragmentary preservation represents a fundamental limitation in palaeoanthropological research, particularly for postcranial elements where symmetry cannot be exploited and large portions of bone are frequently missing. Although a wide range of virtual reconstruction methods exist, many rely on strong anatomical priors, localized interpolation, or reference specimens that risk biasing reconstructions toward predefined morphologies. The present study introduces a new approach for estimating the coordinates of missing landmarks, termed the Reverse Engineering (RE) approach. This method establishes mathematical relationships between morphological patterns observed in complete reference specimens and those preserved in reduced portions of each bone, allowing missing regions to be inferred from patterns of covariation rather than local geometric proximity. We demonstrate that the RE approach performs consistently across a range of reconstruction scenarios, including cases where substantial portions of bone are missing, and in empirical applications to Neanderthal femoral and humeral specimens. Importantly, reconstructed specimens retain morphological signal in subsequent morphometric analyses, regardless of the extent of missing data. While limitations remain, as is the case for all reconstruction methods applied to fragmentary fossil material, the RE approach offers a robust and flexible alternative that does not depend on symmetry or a single reference specimen. As such, it represents a valuable addition to the methodological repertoire available to palaeoanthropologists working with incomplete postcranial remains.

## 1 Introduction

The incomplete nature of the fossil record not only shapes how we perceive and analyze evolutionary trajectories but also influences how we interpret them. Issues related to small sample sizes and sample imbalance are particularly common in palaeoanthropological research. Some species may be represented by only a single or a handful of specimens (e.g., Brunet et al. 1995; Leakey et al. 2001; Haile‐Selassie et al. 2019), whereas others are known from numerous individuals, sometimes restricted to even a single locality (e.g., Arsuaga et al. 1997; Plavcan et al. 2005; Berger et al. 2015).

Beyond the rarity of certain fossils, an equally significant challenge is their fragmentary nature. Taphonomic processes, including both biotic and abiotic agents, frequently distort and damage fossil materials through various mechanisms, leading to the recovery of only partial specimens (e.g., Johnstone 2001; Clarke 1998; Domínguez‐Rodrigo et al. 2013). This can result in considerable alterations to their original morphology, rendering certain features unobservable or displacing key structures from their original anatomical positions. Pre‐depositional processes such as abrasion, polishing, and rounding due to sediment or fluvial transport, as well as consumption and modification by scavengers, contribute to this deterioration. Diagenetic and biostratinomic processes including plastic deformation, chemical dissolution, mineral replacement, and the formation of concretions, further impact preservation. Similarly, post‐exhumation processes can result in sullegic and trephic modifications, which may have an effect on bone structure on both micro‐ and macroscopic scales (Lyman 1994; *inter alia*).

Fragmentation and distortion also introduce significant diagnostic challenges. Extremely small or heavily altered fragments may not only obscure species identification but also make it difficult to determine whether multiple fragments belong to the same individual. This is particularly challenging in contexts with multiple species (see reviews in Grine et al. 2022; Antón and Middleton 2023).

Taken together, these factors present major obstacles in palaeoanthropology, limiting our ability to reconstruct past phylogenetic relationships, biomechanical adaptations, anatomical structures, and even aspects of behaviour. As fossil preservation becomes increasingly compromised with geological age, these challenges become more pronounced, particularly when studying deeper time periods where well‐preserved specimens are rare.

The study of the postcranial skeleton, and more specifically of limb long bones, is particularly affected by these issues, especially when missing some of the most informative areas for the identification of both biomechanical and taxonomic traits: the epiphyses (Johanson, Lovejoy, et al. 1987; Richmond et al. 2020; Aramendi et al. 2024). Considering the wide variability of tissue density throughout long bones, these anatomical elements are highly susceptible to intra‐specimen differential preservation, often leaving only certain portions of the diaphysis intact. Even so, in many cases, specimens may not preserve the precise same portion of the diaphysis or epiphyses, making comparisons with other fossil individuals particularly difficult.

Missing data is a fundamental concern for all types of data analyses, across all fields of science. Nevertheless, when dealing with extant species, these issues become increasingly prevalent. One of the most renowned techniques used to study specimens in paleontology, biology, and the related fields in anthropology, is the use of landmark‐based Geometric Morphometrics (GMMs). Homology is a fundamental component of landmark‐based GMMs, both from a theoretical, practical and mathematical perspective (Bookstein 1984; Oxnard and O'Higgins 2009; Zelditch et al. 2001). In this context, GMMs rely on first the identification of anatomical features presenting a point‐to‐point correspondence that can be identified across all individuals being studied. Once defined, these points, represented as Cartesian coordinates, are superimposed, facilitating the analysis and subsequent identification of morphological patterns among the sample (Mitteroecker and Gunz 2009; Gower 1975; Rohlf and Slice 1990). In the case of fragmentary specimens, however, maintaining this point‐to‐point correspondence is often impossible.

To address this issue, many studies on long bones employing landmark‐based approaches are forced to restrict their analyses to only the landmarks present in all specimens, ensuring comparability, or to very specific bone portions (Almécija et al. 2013; Arias‐Martorell et al. 2023; Tallman 2012, 2013, 2014). However, this approach often results in a significant loss of valuable morphological information, also limiting the comprehensive study of long bones.

Throughout the years a number of approaches have been proposed for the purpose of estimating the position of missing landmarks. Nevertheless, no single approach is free of key disadvantages that hinder these analyses. The objective of the present study is to propose a different means of estimating missing landmark data that performs well where other algorithms may not, such as the prediction of large portions of bone. Nevertheless, as with any predictive model, it is always useful to consider the advantages and disadvantages for each, focusing on the specific task at hand.

### 1.1 State of the Art

Computers have played a considerable role in research related to the virtual reconstruction of specimens. These range from studies that align or piece back the pieces of the puzzle virtually, using 3D models and techniques such as computer tomography (CT) (Zollikofer et al. 1995, 2005), to more statistically oriented results for the estimation and prediction of missing parts (Gunz et al. 2009; Torres‐Tamayo et al. 2025), or correcting for deformation (Schlager et al. 2018; Amano et al. 2022). A large amount of research exists throughout science presenting means of overcoming missing data problems; however, in this study we will focus specifically on the prediction of missing landmarks.

As opposed to many other fields of science, where missing data can often be modeled stochastically, missing landmarks are not independent events. Instead, the probability of a missing landmark is influenced by the absence of neighboring landmarks due to the inherent spatial autocorrelation in morphological structures. This interdependence fundamentally shapes the choice of imputation methods, as missing data cannot be treated as random but must be estimated within the structural constraints of the specimen.

In palaeoanthropology, this typically means that reconstructions are based on a series of strong prior assumptions (Gunz et al. 2004, 2009). These are often based on geometric, mechanical, and biological knowledge, and can be related to a number of factors such as allometry, symmetry, and morphological integration. Previous research relies heavily on a number of these components, particularly symmetry (Ogihara et al. 2006; Gunz et al. 2009; Schlager et al. 2018). In specimens exhibiting bilateral symmetry, coordinates from one side can be projected onto the opposite side to estimate missing landmarks. This procedure is often followed by a correction step, typically using Thin Plate Splines (TPS), to refine landmark positions (Benazzi et al. 2009). Many anatomical elements, such as parts of the appendicular skeleton, however, do not present bilaterally symmetrical features that can be used for this purpose.

Beyond symmetry, TPS‐based approaches are also employed through what are often termed geometric reconstructions, in which a complete reference specimen (or a consensus configuration) is used to estimate missing landmarks in an incomplete specimen. In this context, missing landmarks are first projected into space relative to preserved landmarks and subsequently optimized by minimizing the bending energy between the reference and target configurations (Gunz et al. 2009). Conceptually, this process is analogous to the estimation of semilandmark positions prior to sliding (Gunz 2005). However, such reconstructions rely heavily on assumptions regarding the suitability of the chosen reference specimen, and the accuracy of the estimation is sensitive to both anatomical proximity and the extent of missing data.

Even when symmetry is absent, TPS interpolation can yield high accuracy in controlled simulation studies, particularly when reconstructing features defined by smooth curvature and when missing landmarks are located close to preserved data (Gunz et al. 2009; Arbour and Brown 2014). Nevertheless, TPS suffers from a fundamental limitation: the interpolation function can only reliably estimate morphology in regions constrained by existing observations. As the size of the missing region increases, or as missing landmarks become spatially clustered, prediction accuracy degrades rapidly (*ibid*). From this perspective, the reconstruction of large portions of bone using purely geometric interpolation is expected to result in substantial error.

An alternative family of approaches is commonly referred to as statistical reconstruction, which relies on modeling the patterns of variation and covariation present in a reference sample of complete specimens. These methods typically employ regression‐based frameworks to estimate missing landmark coordinates from preserved landmarks (Neeser 2007; Neeser et al. 2009), exploiting morphological integration across the structure. Nevertheless, originally, methods relied a lot on the use of Mean Substitution (MS), whereby the mean position of landmarks from a consensus is used to predict the location of the missing point. Although the general principle is shared across studies (Neeser et al. 2009; Brown et al. 2012; Arbour and Brown 2014), the specific implementations vary widely, ranging from multiple linear regression to more sophisticated probabilistic approaches such as Bayesian Principal Component Analysis (BPCA). In many cases, statistical reconstruction methods have been shown to outperform geometric interpolation, particularly when deformations are slightly larger and are not locally constrained.

However, statistical reconstruction methods rely critically on the availability of sufficiently large and representative reference samples to accurately model variance–covariance structure (Neeser et al. 2009). Moreover, because these methods explicitly exploit covariance during estimation, their use may influence downstream analyses that rely on the same covariance structure, such as principal component analysis or regression‐based comparative studies. As a result, method choice must be carefully matched to both the nature of the missing data and the intended analytical use of the reconstructed specimen.

Recent advances further extend these statistical approaches through the development of multi‐scalar symmetrical retrodeformation procedures, and Statistical Shape Modeling (SSM). In the first of these approaches, Schlager et al. (2018) formulate retrodeformation as a symmetrisation problem, using a closed‐form solution that first symmetrises local neighborhoods of bilateral semilandmarks, and then solves for global symmetry while minimizing deformation in each local region (Ghosh et al. 2010). To address the issue of asymmetric noise inherent in semilandmark placement, the approach includes a regularization step in which semilandmarks are allowed to slide along the surface to minimize bending energy relative to a perfectly symmetric configuration, thereby ensuring that only meaningful asymmetry is corrected. The resulting transformation is applied to the entire 3D mesh using TPS interpolation, enabling more accurate and robust restoration of fossil morphology, especially in cases where anatomical landmarks are sparse or unevenly distributed.

SSM, on the other hand, integrates dense geometric data with covariance‐based prediction frameworks. Notably, Torres‐Tamayo et al. (2025) demonstrated the successful application of SSM to the reconstruction of large, complex missing anatomical regions in hominin pelves, particularly the pubis. This approach combines traditional landmarks with extensive curve and surface semilandmarks to achieve high geometric resolution and constructs predictive models by decomposing the covariance structure of aligned three‐dimensional surface data using principal component analysis. Their results indicate that SSM provides greater robustness than TPS when reconstructing large missing regions, particularly by reducing extreme prediction errors.

Importantly, this work reinforces earlier findings that reference sample size and variance–covariance structure are often more important than strict taxonomic proximity. Models incorporating multiple extant species outperformed those relying on a single taxon, highlighting the value of broad morphological sampling when reconstructing fossil material (Torres‐Tamayo et al. 2025).

Taken together, existing reconstruction methods reveal a trade‐off between geometric assumptions, statistical power, and robustness to extensive missing data. While geometric approaches such as TPS remain valuable under specific conditions, and statistical reconstruction methods perform well when reference data are abundant, the reconstruction of large missing portions of fossil specimens remains a substantial challenge. These limitations motivate the development and evaluation of alternative predictive frameworks capable of leveraging both morphological integration and modern computational approaches.

## 2 The Reverse Engineering Approach

The term “Reverse Engineering” (RE) in our approach stems from the idea of deconstructing a dimensionality reduction technique to uncover the underlying patterns it encodes. Similar to how TPS is used to visualize shape transformations across dimensions, our approach seeks to leverage these types of visualizations for a different purpose; estimating landmarks based on morphological patterns. For this to succeed, it is essential to define a reduced‐dimensional feature space that captures the maximum amount of morphological variability. In this context, we define a feature space as a multidimensional mathematical space in which observations are represented by their measured characteristics (features), with each feature defining one dimension of the space. Unlike other missing landmark estimation approaches that rely on a reference specimen for projecting landmarks, the RE approach seeks to generalize morphological variations across samples and populations, avoiding constraints tied to specific species and/or groups. For this reason, the taxonomic attribution and sex of the individual is not as relevant; during the reconstruction we pool all species and sub‐groupings together, without differentiation. Nevertheless, in order for this approach to be reliable, the analyst must attempt to include a reference sample of multiple species that captures as much of the natural variability or morphological patterns as possible. The reference sample must, however, be constrained to taxa that are phylogenetically and morphologically related to the specimens that require reconstruction (e.g., use of great apes to reconstruct hominins) to avoid feature spaces that introduce unnecessary morphological noise. The reference sample used will therefore depend completely on each individual case study, and the types of research questions being asked.

### 2.1 Mathematical and Theoretical Definition

Any set of landmark coordinates, *X* ∈ *ℝ* ^*p×k*^, where *p* is the number of landmarks, and *k* is the number of dimensions, can be described in a lower‐dimensional space using dimensionality reduction techniques. One of the most widely used approaches in GMM is Principal Component Analyses (PCA). PCA decomposes the covariance matrix of *X* through eigenvalue decomposition, yielding a set of eigenvectors and eigenvalues (Jollife 2002). These eigenvectors define the principal axes, while eigenvalues quantify the variance along each axis. By projecting *X* onto the eigenvectors, PCA identifies the most significant axes corresponding to directions of maximum variance in the data, resulting in a new feature space defined by the principal components.

Dimensionality reduction is then achieved by selecting a subset *d* of these principal components, *z* ∈ ℝ^*d*^, focusing on those that explain the most variance. This retains the most meaningful information while reducing complexity in a lower dimensional feature space. Although there is no universal consensus on the optimal value for *d*, this parameter can be tuned and tested using different criteria to obtain the best results (Jollife 2002; Courtenay et al. 2024a).

**FIGURE 1:**
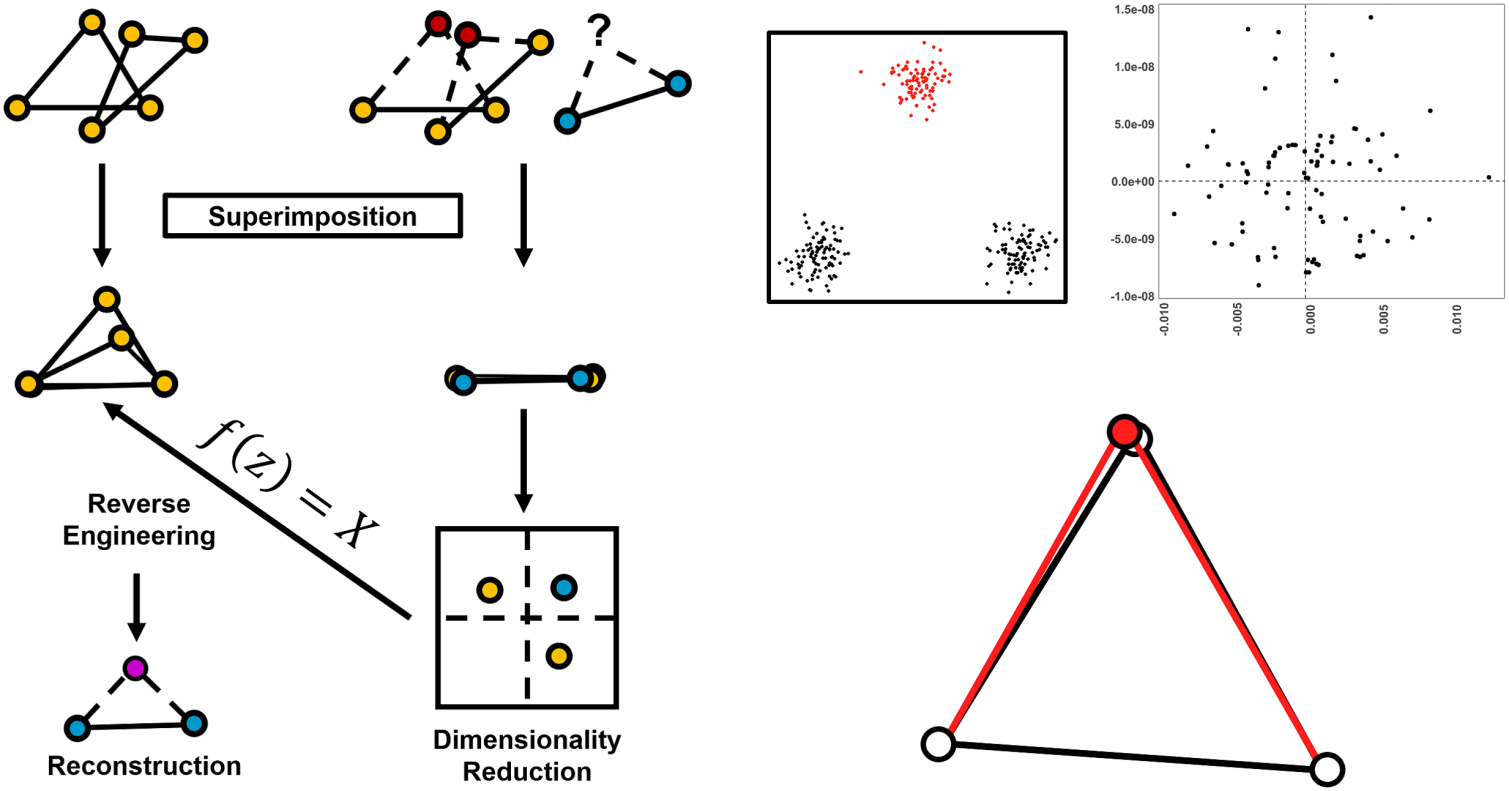
Schematic figure describing the basic concepts behind the Reverse Engineering approach, alongside an example using theoretical data for the reconstruction of the missing tip of a triangle. In the left hand panels, we have the Procrustes coordinates describing a set of approximately equilateral triangles, the PCA feature space describing the bottom left and right landmarks only, and the final predicted morphology of a triangle missing its tip. Please note this is just a figurative representation of the workflow, and does not account for any potential contamination that is discussed in Section 2.1. In the right hand panel, we present a simple example of reconstructing the tip of a triangle. We begin with the landmark coordinates of an entire set of triangles, we extract the PC scores that describe the morphological descriptors of these triangles, and use this to predict the tip of a new triangle. The lower section presents the original triangle (black) and the reconstructed triangle (red), showing a relatively low error in the final reconstruction.

The RE approach seeks to find a mathematical function, *f* (*z*) *= X*, that maps our morphological descriptors, *z*, to the landmark coordinates of complete specimens, *X* (Figure 1). This enables the estimation of missing landmark coordinates by leveraging information only from the subset of landmarks that are consistently present across all specimens, *X′*. The process of determining *f* (*z*) can be naturally framed as a regression problem that we can solve using supervised approaches.

The simplest form of RE is the linear approach, where *f* (*z*) is defined as a multivariate linear regression, such that (Equation 1).

(1) $$f\left(z\right)=\vec{z}\vec{\beta }+\alpha$$

where *β* represents the slope, and *α* the *y*‐intercept of the regression line. Here the regression parameters *α* and *β* are estimated using Ordinary Least Squares (OLS) optimisation (Hastie et al. 2009). Alternatively, these can also be computed using Stochastic Gradient Descent (SGD) (Robbins and Monro 1951; Rumelhart et al. 1986), depending on the needs of the analyst; nevertheless, for simplicity, OLS is more than sufficient for this task.

For the purpose of performing missing landmark estimation, we take all the complete specimens from our referential sample and compute *z* only on *X′*, containing only those landmarks that are also present in the incomplete specimen we wish to reconstruct. We then estimate the regression parameters *α* and *β* from the relationship between *z* and the complete specimens *X* and use these parameters to predict the coordinates of the missing landmarks.

To evaluate the performance of this approach, we use cross‐validation. Given that most palaeoanthropological datasets are relatively small, we aim to leverage the available data as effectively as possible to define *f* (*z*) and reconstruct the incomplete specimen. To achieve this, we employ a Leave‐One‐Out Cross‐Validation (LOOCV) strategy, which involves fitting the model on all but one specimen in the dataset and using the left‐out specimen for evaluation. This ensures that every specimen is used for both training and validation. The error for *f* (*z*) is then calculated by computing the square root of the Mean Squared Error (RMSE) between the predicted and original coordinates for each specimen in our referential dataset.

It is crucial, however, to take a moment to discuss data leakage and the validity of performing predictive tasks on data derived from GMMs. When evaluating the performance of predictive algorithms in Deep and Machine Learning (DL & ML), the separation between training, testing, and validation datasets must be absolute to avoid contamination (Bishop 1991, 1995, 2006; Goodfellow et al. 2016). Data leakage, whether intentional or inadvertent, undermines the validity of reported accuracies. This issue has been identified in several recent archaeological studies employing ML and DL approaches, leading to erroneous results (Calder et al. 2022; Courtenay et al. 2024a).

One potential source of data leakage that has been identified in GMM studies is the use of PC scores in predictive modeling, particularly if the train/validation split is conducted after computing the PC scores. Since eigenvalues and eigenvectors are derived from the entire dataset—including the validation set—this introduces contamination, as noted by Calder et al. (2022), and later acknowledged by Courtenay et al. (2023). To prevent this, train/validation splits must occur before computing PC scores. The validation set is then projected onto the feature space defined by the training set's eigenvalues and eigenvectors.

An additional unique challenge in GMMs involves data leakage from the Generalized Procrustes Analysis (GPA) used for superimposing landmarks (Courtenay 2026). GPA is an iterative process that uses a central configuration (Rohlf and Slice 1990), typically the mean shape, to align individuals. Since this central configuration is calculated from the entire dataset, including the validation set, we could argue that here too lies a potential source of data leakage. Unlike PC score‐based contamination, this issue is more complex due to the fundamental role GPA plays in GMM data processing.

Naturally, the definition of *f* (*z*) can be much more complicated than the simple linear regression model presented in Equation (1), depending on the task at hand. For this purpose, a number of additional regression algorithms can also be considered that are typical to the fields of DL and ML. These include, but are not exclusive to, the training of Robust Linear Regression models (RLRs), linear Support Vector Machines (SVMs), as well as other non‐linear algorithms such as Decision Trees (DTs).

**FIGURE 2:**
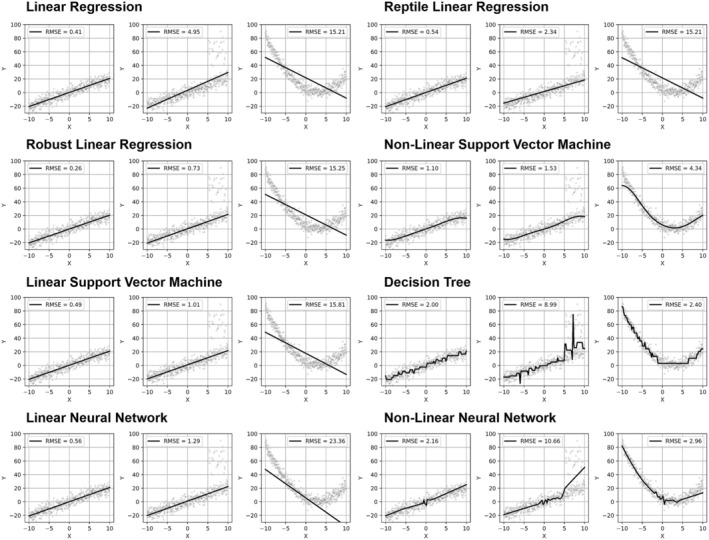
A visual demonstration of how different algorithms can be used for the purpose of solving three different types of regression problems. The first column for each algorithm presents a basic regression problem, where *y* = 2*x*, while the second column presents the same dataset with the inclusion of outliers and noise toward the higher values of *x* so as to test for algorithm robusticity. The final example presents a non‐linear relationship between the *x* and the *y* value, such that y=12x2−3x+5. In all examples the RMSE presents the distance between the theoretical line and the proposed line by each algorithm.

Visual demonstrations about how these algorithms function for different challenges in regression problems can be seen in Figure 2.

RLR is an extension of the standard linear regression that is designed to handle datasets with outliers or violations of normality assumptions. RLR does this by minimizing the influence of extreme values on the model, using an Iterative re‐Weighted Least Square (IWLS) optimisation algorithm, as opposed to the traditional OLS approach. The weights in RLR are determined based on Huber's ψ function (Huber 1981; Hampel et al. 1986), whereby large residuals are down‐weighted giving them less influence on the estimation of *α* and *β* parameters, while small residuals are rewarded. This helps ensure the final regression model is less sensitive to noise or outliers.

SVMs are powerful and popular models that define *f* (*z*) within a margin of tolerance, often referred to as the *ϵ*‐tube (Cortes and Vapnik 1995; Drucker et al. 1997). This tube allows the model to ignore deviations within a maximized margin, focusing instead on minimizing prediction error for points outside this region of size *ϵ*. This is defined by a cost parameter, *c*, which governs the trade‐off between model complexity and the penalty applied to deviations outside the margin, ensuring robustness to outliers while maintaining generalization. While SVMs can incorporate kernel functions to capture non‐linear relationships, in the present study we found linear models to work best.

DTs are non‐linear algorithms that partition the feature space into distinct regions by iteratively splitting the data based on feature thresholds (Breiman et al. 1984). These splits are determined by a splitting criterion, such as variance reduction, which aims to minimize the variability of the dependent variable within each region. Within each partitioned region, the model fits regression lines or assigns predicted values, resulting in a piecewise approximation of the target function. DTs are well suited for capturing complex, non‐linear patterns in data.

The downside of RLRs, DTs and SVMs, however, lies in their inability to produce multivariate outputs; they are only able to predict one variable at a time. This implies that the algorithms have to be trained for each coordinate value of each landmark separately, implying an increase in computational costs, unlike traditional linear models that are able to handle both multivariate inputs as well as outputs.

### 2.2 Metalearning

Metalearning (MtL) is a branch of artificial intelligence dedicated to the development of algorithms that are more generalisable to different tasks (Andrychowicz et al. 2016). Unlike traditional ML, where a model is trained on a single task, MtL aims to enable models to learn from a variety of tasks and adapt quickly to new, unseen tasks with minimal data. By leveraging experience from previous tasks, MtL algorithms attempt to discover more efficient strategies for learning, such as identifying useful parameter initialisations or learning representations that are applicable across different tasks. This idea of learning optimal strategies for learning is often referred to as “learning how to learn”, hence the prefix “Meta” in “Metalearning”. Popular MtL approaches include techniques such as Meta‐SGD (Andrychowicz et al. 2016; Li et al. 2017), which optimises the process of gradient descent itself, and Model‐Agnostic Metalearning (MAMtL) (Finn et al. 2017), which seeks parameter initialisations that enable rapid adaptation to new tasks. These methods are unified by their emphasis on improving model initialisation, allowing faster convergence with fewer gradient steps on related tasks (Finn et al. 2017; Dauphin and Schoenholz 2019). In the present study, we adopt the Reptile algorithm, a computationally efficient MtL strategy (Nichol et al. 2018).

MtL, for example, can be used as a different strategy to OLS and IWLS for the calculation of *α* and *β* parameters in Equation (1). For this purpose, let *T*_*i*_ represent a distinct yet related subset of data within the set *T* ∈ {*T*_*1*_, …, *T*_*t*_}, where *t* is the total number of subsets, all of which share a common set of regression parameters, denoted as *θ* = {*α*, *β*}. These subsets are often referred to as tasks. The learning process begins with *θ* initialised randomly. The model is then trained on each task individually, minimizing the loss function *L* specific to *T*_*i*_ using SGD. This results in an optimized set of parameters for each task, denoted as *θ'*_*i*_. To generalize this across all of *T*, we then perform a meta‐update, which aggregates the parameters *θ'*_*i*_ specific to *T*_*i*_, and refines the shared parameter *θ* used to initialise the training process. In Reptile, this is achieved by updating *θ* toward the average of the task‐specific parameters, such that (Equation 2).

(2) $$\theta \leftarrow \theta +\varepsilon \cdot \frac{1}{t}\sum_{i=1}^{t}\left(\theta_{i}^{\prime }-\theta \right)$$

where *ϵ* is the meta‐learning rate controlling the magnitude of updates. This process leverages the relationship among related tasks to improve the initialisation of *θ*, making the learned parameters more effective for new, unseen tasks. This therefore allows the model to generalize across tasks by capturing shared structures in the data, leading to more robust parameter estimates.

### 2.3 Neural Networks

The final set of algorithms considered for the purpose of regression were Neural Networks (NNs); a set of models that are built of multiple layers of mathematical functions, known as neurons (Bishop 1995; Goodfellow et al. 2016). These neurons can be adapted to map both linear and non‐linear relationships between variables. Mathematically, a neuron in any layer of an NN computes the sum of its inputs, *x*, multiplied by a set of weights, *w*, with the addition of a bias term, *b*. From this perspective, *w* and *b* can be considered analogous to *β* and *α* from Equation (1), respectively. The advantages of NNs, however, stem from the inclusion of an activation function to which the output of the neuron is passed, and from the ability to stack multiple layers of neurons, enabling the model to define hierarchical relationships between variables (Goodfellow et al. 2016). Activation functions are the fundamental means by which non‐linearity can be established in the NN; however, for the present study, optimal results were obtained using simple linear functions.

## 3 Materials and Methods

### 3.1 Primate Long Bone Dataset

For the purpose of evaluating RE as a missing landmark estimation strategy, we used a dataset consisting of a total of 400 long bones from extant great apes, including the humeri and femora of individuals from the genera *Homo, Gorilla, Pan*, and *Pongo*.

Only specimens without external signs of pathologies or bone deformation, belonging to adult individuals of both sexes, were selected from various collections across Europe (Table 1). The bones were digitized using an Artec Space Spider and post‐processed with an accuracy of 0.15 mm. Additionally, 3D models of extant great apes meeting the aforementioned criteria were downloaded from Morphosource (Department of Vertebrate Zoology, National Museum of Natural History, Smithsonian Institution; see more details in Almécija et al. (2024)), the New Mexico Decedent Image Database (NMDID; Edgar et al. 2020), and the former Digital Morphology Museum KUPRI website (Primate Research Institute, Kyoto University). These data consisted of surface scans, rendered 3D volumes from CT scans, or raw CT files. In the latter case, bones were post‐processed in Avizo 2020.3 (Thermo Fisher Scientific) to generate 3D models through semi‐automated segmentation, whereby most of the segmentation was performed using available automatic tools and subsequently refined manually when needed. When necessary, bones were translated and mirrored in Avizo to standardize them as right humeri and femora.

**TABLE 1:** Extant great ape sample used in this study to train the models.

Group	*N*	Sex	Species	Collection^a^
*Homo*	50 humeri 50 femora	Male = 17; Female = 13; Unknown = 20 Male = 16; Female = 14; Unknown = 20	*Homo sapiens*	Duckworth NMDID
*Pan*	50 humeri 50 femora	Male = 22; Female = 23; Unknown = 5 Male = 24; Female = 26	*Pan troglodytes* (32) *Pan paniscus* (18)	KUPRI MCNB Morphosource NMW RMCA
*Gorilla*	50 humeri 50 femora	Male = 21; Female = 18; Unknown = 11 Male = 25; Female = 18; Unknown = 7	*Gorilla beringei* (25) *Gorilla gorilla* (25)	KUPRI Duckworth MCNB Morphosource NMW RMCA
*Pongo*	50 humeri 50 femora	Male = 17; Female = 20; Unknown = 13 Male = 19; Female = 19; Unknown = 12	*Pongo pygmaeus* (29) *Pongo abelii* (21)	Duckworth KUPRI MCNB MorphoSource NMW UZ

^a^ Collections: Duckworth = Duckworth Collection in Cambridge (UK); KUPRI = Digital Morphology Museum KUPRI (Japan); MCNB = Museu de Ciències Naturals de Barcelona (Spain); NMDID = New Mexico Decedent Image Database NMW = Naturhistorisches Museum Wien (Austria); RMCA = Royal Museum for Central Africa (Belgium); UZ = Evolutionary Anthropology Collection of the Universität Zürich (Switzerland).

Both humeri and femora were landmarked using 40 fixed anatomical landmarks (Table 2), primarily located on the epiphyses and metaphyses of the long bones. A network of 160 surface semilandmarks was projected and slid on the shaft of each element, using 8 points (four on the most proximal and most distal sections) as fixed points to constrain the sliding process. Landmarking, semilandmaking and sliding were performed using the EVAN ToolBox. For detailed information on the landmarking process see the File S1.

**TABLE 2:** Anatomical landmarks used to describe the humerus and femur sample.

*N*	Landmark description for the humerus	Landmark description for the femur
1	Most projecting point of the lateral epicondyle	Most proximal point on the femoral head
2	Proximal intersection point between the lateral epicondyle and the capitulum	Proximal point of the femoral head perimeter
3	Distal intersection point between the lateral epicondyle and the capitulum	Posterior point of the femoral head perimeter
4	Most projecting point on the lateral trochlea	Distal point of the femoral head perimeter
5	Point of maximal curvature on the trochlear groove	Anterior point of the femoral head perimeter
6	Most projecting point on the medial trochlea	Trochanteric fossa
7	Proximal intersection point on the medial trochlea	Surface of the quadrate tubercle
8	Most projecting point of the medial epicondyle	Point of maximal curvature of the femoral neck
9	Proximal anterior point of the lateral trochlea	Intersection point between pectineal line and linea aspera
10	Proximal anterior point of the medial trochlea	Most prominent point of the lesser trochanter
11	Proximal posterior point of the lateral trochlea	Midpoint of intertrochanteric crest
12	Proximal posterior point of the medial trochlea	Most projecting point of the lateral epicondyle
13	Proximal point of the olecranon fossa	Most projecting point of the medial epicondyle
14	Distal point of the olecranon fossa	Distal point on the lateral epicondyle
15	Lateral point of the olecranon fossa	Distal point on the medial epicondyle
16	Medial point of the olecranon fossa	Midpoint of femoral condyles
17	Point of maximal curvature between the medial epicondyle and the shaft	Intercondylar fossa
18	Proximal most projecting point of the lateral supracondylar ridge	Medial peak of the patellar surface
19	Proximal point of the lateral supracondylar ridge	Lateral peak of the patellar surface
20	Proximal end of the ventral segment of the deltoid tuberosity	Intersection point between the patellar Surface and the lateral epicondyle
21	Distal end of the ventral segment of the deltoid tuberosity	Intersection point between the patellar surface and the medial epicondyle
22	Distal end of the dorsal segment of the deltoid tuberosity	Proximal point of the lateral condyle
23	Proximal end of the dorsal segment of the deltoid tuberosity	Lateral point of the lateral condyle
24	Distal point of the greater tubercle crest	Medial point of the lateral condyle
25	Distal point of the lesser tubercle crest	Proximal point of the medial condyle
26	Distal end of the outline of the infraspinatus insertion	Lateral point of the medial condyle
27	Lateral end of the outline of the infraspinatus insertion	Medial point of the medial condyle
28	Proximal end of the outline of the infraspinatus insertion	Adductor tubercle
29	Medial end of the outline of the infraspinatus insertion	Intersection point between medial and lateral supracondylar lines
30	Anterior end of the outline of the supraspinatus insertion	Midpoint on the lateral supracondylar ridge
31	Intersection point between the humeral head perimeter and long head of the biceps brachii	Midpoint on the medial supracondylar ridge
32	Proximal end of the outline of the subscapularis insertion	Distal end point of the intertrochanteric line
33	Lateral end of the outline of the subscapularis insertion	Proximal point of the fovea
34	Distal end of the outline of the subscapularis insertion	Posterior point of the fovea
35	Medial end of the outline of the subscapularis insertion	Distal point of the fovea
36	Proximal point of the humeral head perimeter	Anterior point of the fovea
37	Posterior point of the humeral head perimeter	Proximal point of the greater trochanter
38	Distal point of the humeral head perimeter	Posterior point of the greater trochanter
39	Anterior point of the humeral head perimeter	Distal point of the greater trochanter
40	Midpoint of the humeral head	Anterior point of the greater trochanter

In addition to the extant sample, the 3D models of two fairly complete Neanderthal specimens, one femur (Neanderthal 1, Germany) and one humerus (Simanya SI‐1, Spain), were landmarked following the above‐described protocol. The Neanderthal 1 (Fuhlrott 1859) mesh was obtained from a cast in the Duckworth collection using the Artec Space Spider, and all landmarks and semilandmarks could be identified. The Simanya humerus 3D model (Morales et al. 2023) was provided by Antonio Rosas and Juan Ignacio Morales and could be landmarked using 198 out of the 200 points used for the rest of the specimens. Due to preservation issues, the landmarks corresponding to the distal and lateral ends of the outline of the infraspinatus insertion could not be identified (LM 25 and 26, Table 2). These two specimens were not included in the training set but were used solely to assess the method's ability to reconstruct “unknown” morphologies. This evaluation is crucial for applications in palaeoanthropology and paleontology, where fossil specimens exhibit unique features that may not be represented in extant groups.

### 3.2 Missing Landmark Estimation and Method Validation

For the purpose of evaluating the RE approach, we performed a number of simulations on our referential dataset, removing varying numbers of landmarks and reconstructing them so as to assess the accuracy of different regression models on the reconstruction of the bones. These simulations consisted of 6 trials per bone, where all landmarks were deleted on (1) the proximal epiphysis, (2) the distal epiphysis, (3) the proximal and the distal epiphysis, (4) the diaphysis, (5) the diaphysis and proximal epiphysis, and (6) the diaphysis and distal epiphysis.

For each of these trials, the remaining landmarks were then superimposed using GPA in form space, followed by dimensionality reduction using PCA. GPA can be performed either for the study of *shape*, including the scaling of landmark coordinates to remove the effects of size, or *form* (shape + size). Because GPA without scaling preserves centroid size, form space was used so that the corresponding calculation of errors could be reported in millimeters, providing a more interpretable estimation of reconstruction error. To perform RE on these feature spaces, the number of selected Principal Components (PCs) used as morphological descriptors was adjusted to achieve the lowest possible error in subsequent reconstructions. Nevertheless, in all examples, the minimum number of PCs selected always represented at least 95% of the total morphological variance. Once the morphological descriptors of shared landmarks (*z*) had been selected, we trained the different regression algorithms described in Section 2 to establish the relationship between *z* and the complete set of coordinates (*X*).

Across all examples, we used LOOCV to iteratively calculate the error for all of the reference specimens using each algorithm. LOOCV involves systematically removing one individual, training regression algorithms on the remaining individuals, and then validating the algorithm's accuracy by predicting the missing landmarks of the removed specimen, denoted by *S*_*val*_.

To address the challenges imposed by Procrustes contamination for validating our RE approach, we ensured that *S*_*val*_ was excluded before GPA was performed. This precaution avoids contamination during superimposition and the definition of *X* in *f* (*z*) = *X*. This is also more analogous with what would happen in real‐world applications for the reconstruction of fossils, where *X* would not be able to be defined by including the fossil specimen. This, however, creates an additional challenge: the predicted specimen, having bypassed the initial GPA, will fall in a different coordinate space from the original; therefore, direct calculation of RMSE is no longer possible. To resolve this, we have to perform a secondary GPA in form space to align the *S*_*val*_ with both *X* and the predicted coordinates of the validation specimen, *S*_*val*_ *'*, so that all coordinates fall in the same coordinate space as *X*. Following this alignment, we can calculate the RMSE between *S*_*val*_ and *S*_*val*_ *'*, which would technically be analogous with calculating the Procrustes distance in form space from the original individual to the reconstructed individual.

To ensure reproducibility and clarity, the workflow of evaluating RE is described in File S2 using pseudocode, outlining the step‐by‐step procedure in a computationally explicit manner, typical in ML and DL research.

Once RMSE values had been calculated for each individual, descriptive statistics were then used to evaluate the overall error of each approach, using the median to calculate the central tendency, and 95% confidence intervals computed using interquantile ranges.

Following this, PCAs on both the reconstructed and the original complete specimens were performed, similar to the evaluation approach described by Gunz et al. (2004). In this approach, PCAs are presented using arrows where the beginning of each arrow indicates the individuals' original position in feature space, while the tip of the arrow indicates their new position after reconstruction. For these analyses, reconstructions were performed in both shape and form space.

To then evaluate the performance of algorithms on fossil data, the same approach was performed on the two complete Neanderthal specimens; portions of the bone were progressively deleted, and then reconstructed using the *f* (*z*) algorithms defined on our referential dataset. These evaluations considered the RMSE, as described above, as well as where the specimens fall in the PCA feature space before and after reconstruction.

Once missing landmarks have been estimated, an additional evaluation approach can be considered: the warping of a reference mesh to these newly established coordinates, and calculating the deformation and distance between triangles with the reference mesh and the warped mesh. This provides a visual aid for identifying the areas of greatest differences between the reconstructed and original specimens.

### 3.3 Regression Models

As previously stated, for the purpose of this study we tried and tested a number of different regression models, including simple Linear Regression models (LR), RLRs, DTs, SVMs, NNs, and MtL (Table 3). To assess the value of the RE approach with already existing methods, we compared our results with reconstructions performed by TPS interpolation, *sensu* Gunz et al. (2004, 2009), as well as the BPCA missing value estimator, *sensu* Strauss et al. (2003).

**TABLE 3:** Summary of the regression models used in the study to validate the RE approach.

Method	Abbreviation	Brief description
Linear regression	LR	Fits a linear relationship between input variables and the target outcome.
Robust linear regression	RLR	Linear regression extension designed to reduce the influence of outliers and noisy observations.
Linear support vector machines	SVM	Learns a linear decision boundary that maximizes the margin between classes.
Non‐linear decision trees	DTs	Tree‐based models that recursively partition the feature space to capture non‐linear relationships.
Reptile meta‐learning	MtL	Gradient‐based meta‐learning method that learns model parameters enabling rapid adaptation to new tasks.
Neural networks	NN	Multi‐layer computational models capable of learning complex linear and non‐linear patterns from data.

For algorithms such as LR, RLR and SVM, algorithms were simply trained using their default hyperparameters. No tuning of these parameters was performed, as the standalone algorithms are sufficient to perform simple regression without the need for additional tuning. For DT, however, the only parameter that was adjusted was the maximum depth of trees (depth = 3). This it was found to produce the least amount of overfitting on toy datasets prior to their application on landmark data.

For NNs, a simple model was defined with two hidden layers. The hidden layers were defined to have *h* number of neurons, where *h* is *d +* ⌈ *0.5d* ⌉ (recall from Section 2 that *z* ∈ ℝ^*d*^). In between the first and second hidden layer, a Dropout layer was included with *p* = 0.2. All layers were initialised with a uniform kernel initialiser (*sensu* He et al. 2015), whereas linear activation functions were included for all layers. Finally, the algorithm was trained using the Adam optimiser (Kingma and Ba 2015), as opposed to standard SGD, using Mean Squared Error (MSE) as a loss function. Adam was employed using its default parameters (Learning rate = 0.001, *β*_1_ = 0.9, *β*_2_ = 0.999, *ϵ* = 1 × 10^−7^). Using MSE as opposed to RMSE for optimizing the NN is performed to ensure the model's gradients are easier to compute, proving to be better for optimisation purposes. Nevertheless, final evaluation of the algorithm still used the RMSE, as described in Algorithm 1, File S2, for a more interpretable presentation of the results. NNs were trained for 500 epochs with batch sizes of 10 (see Chollet 2017, for explanations on the different elements of how neural networks are designed and trained). During the training of the NN, an additional 10% of the dataset was set aside for weight optimisation. Although 10% is a relatively small fraction, this choice aimed to maximize the data used for training especially in the context of small palaeoanthropological datasets. Nevertheless, adjusting this parameter was not observed to produce a considerable change in the final results. Likewise, all hyperparameters and the overall architecture of the NN was defined through a large series of random trials to find parameters that produced optimal results. This architecture, however, may require tweaking for specific applications in other studies in the future.

For the purpose of MtL, we trained another NN with the same architecture as described above, using the Reptile algorithm for meta‐optimisation (Nichol et al. 2018). The Adam optimiser was again used, as it performed better than SGD, while the number of hidden neurons and linear activation functions were maintained. In this case, Adam's learning rate was set to 0.01, however *β*_1_, *β*_2_ and *ϵ* were maintained the same. The Reptile algorithm used a meta‐learning rate of *ϵ* = 0.1, while *t* was set to 10, consisting of 30 samples, and then optimised using a mini batch size of 10 for 10 iterations. The overall training loop ran for 200 epochs. As before, MSE was used as the loss function to optimise, while RMSE was used to report final error.

### 3.4 Software

This study employed a mixture of the R (v.4.4.1) and Python (v.3.11.9) programming languages. For geometric morphometrics, the *GraphGMM* library (v.1.0.0; Courtenay et al. 2024b), available on GitHub, was used for the majority of calculations, including GPA, PCA and the visualization of extreme shape changes, while the *geomorph* library (v.4.0.9; Adams et al. 2025) was used to apply TPS missing landmark estimation following the methods outlined by Gunz et al. (2004, 2009), and BPCA was performed using the *pcaMethods* library (v.1.93.0; Stacklies et al. 2025). For the regression models, LR was performed using the *GraphGMM* library (v.1.0.0; Courtenay et al. 2024b), RLR in *MASS* (v.7.3–60.2; Venables and Ripley 2002), SVM in *e1071* (v.1.7–14; Meyer et al. 2025), and DT in *rpart* (v.4.1.23; Therneau and Atkinson 2025).

NNs and MtL Reptile meta‐optimisation were carried out in Python (v.3.11.9), using the Tensorflow (v.2.12.0; Abadi et al. 2015) and Numpy (v.1.23.5; Harris et al. 2020) libraries.

To perform mesh warpings, we used the *geomorph* library; however, mesh‐to‐mesh distance calculations and heat‐maps were computed and visualized in the software CloudCompare (v.2.14). Any additional visualization was performed in JavaScript using the Amcharts (v.4) library.

File S2 contains documented code for the implementation of metalearning regression, as well as the correct evaluation methods.

## 4 Results

### 4.1 Evaluation of the Reverse Engineering Approach

**FIGURE 3:**
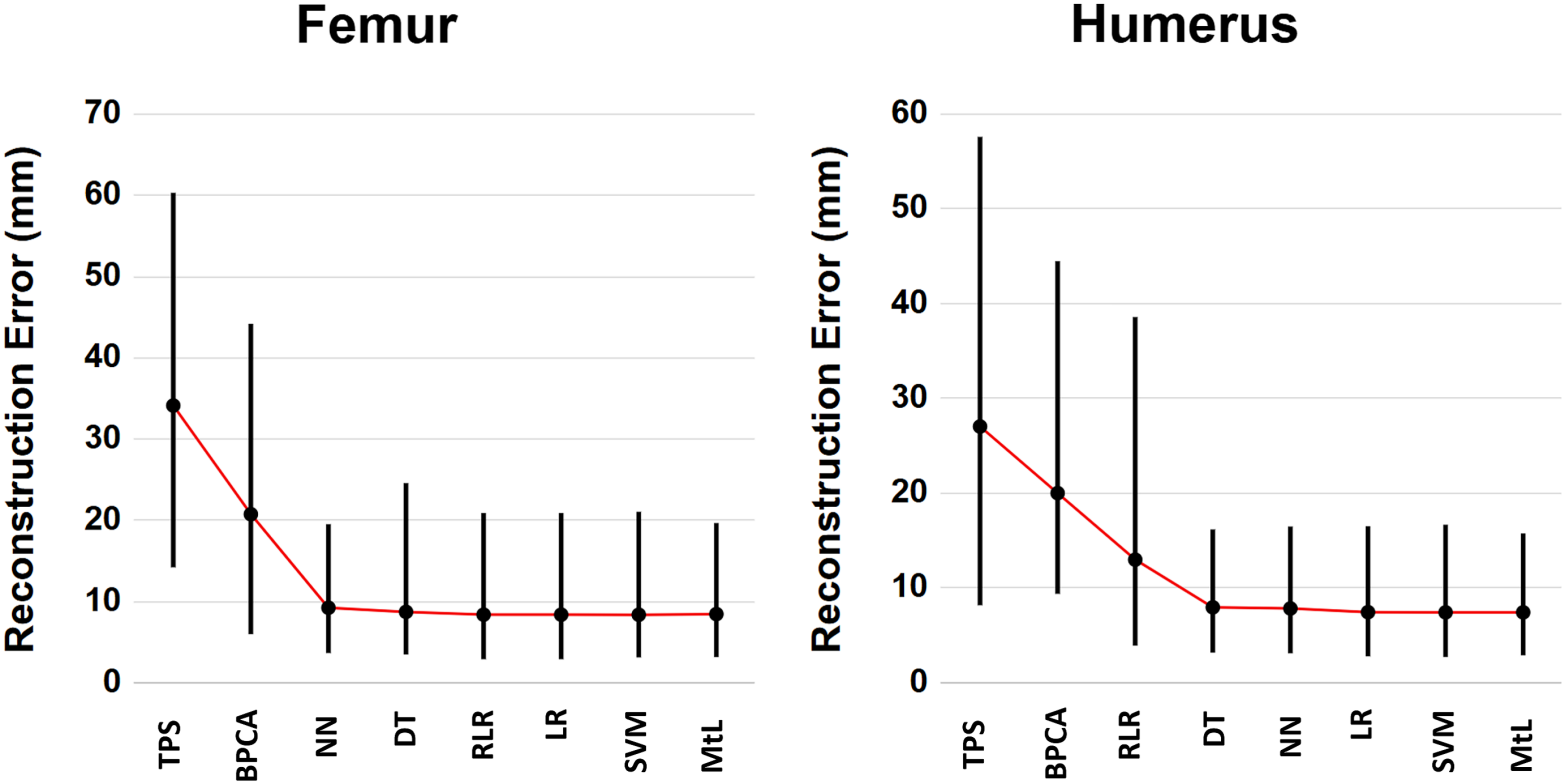
Comparison of different reconstruction techniques and regression models for the reconstruction of the distal epiphysis and diaphysis of the femur and humerus. Algorithms are presented in order of the lowest central tendency of reconstruction errors. BPCA, bayesian principal components analysis; DT, decision tree; LR, linear regression models; MtL, reptile meta‐learning for the optimisation of neural networks; NN, neural network; RLR, robust linear regression; SVM, support vector machines; TPS, thin plate spline interpolation.

**FIGURE 4:**
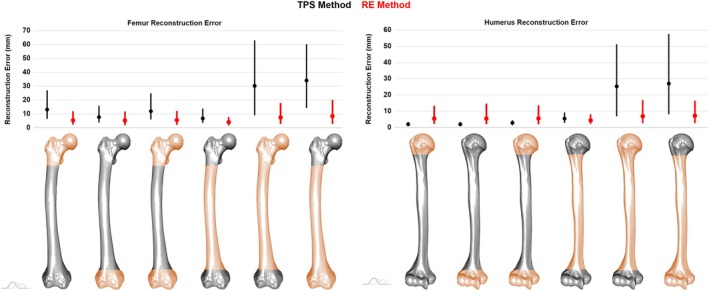
Error plots comparing the Thin Plate Spline and Reverse Engineering using MtL for the estimation of different portions of the bone (shown in orange).

**FIGURE 5:**
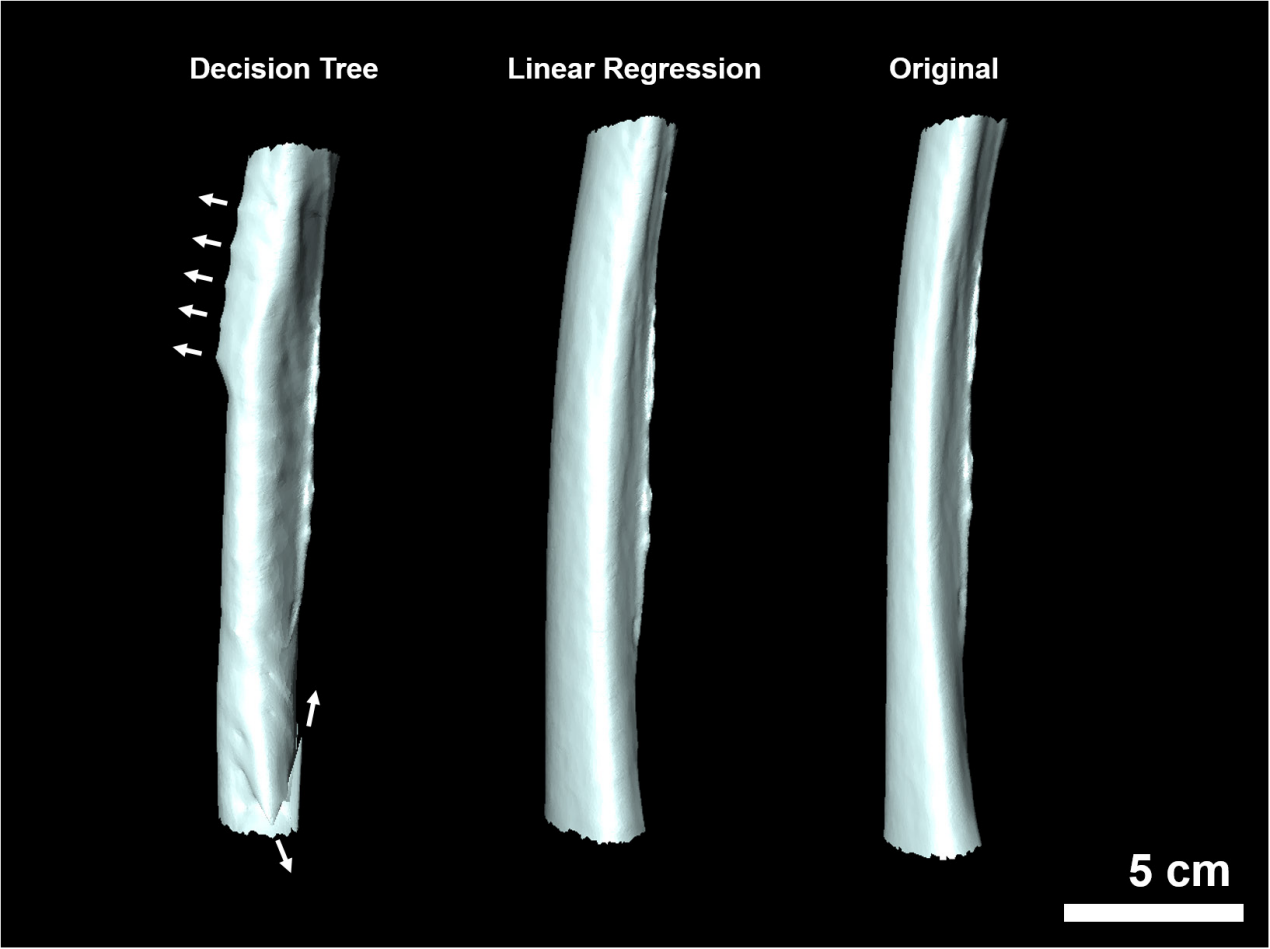
Typical imperfections identified in the reconstructed femoral shaft of a modern human individual using decision tree regression models, in comparison with reptile metalearning‐trained neural networks. Arrows indicate where landmarks have displaced considerably from the rest of the landmark configuration, identifying the need to use regression algorithms that are able to model the spatial relationship between the predicted landmarks.

For all proportions of missing data, the RE approach proves to be an effective means of predicting the position of missing landmarks when using regression models that support multivariate outputs. Across all methods, the MtL is generally found to perform the best (Figure 3), even if marginally, especially for considerably large portions of bone that require reconstructing (Figure 4, Table 4). Nevertheless, the traditional LR model also performs well for many cases, exhibiting a lower 95% confidence interval that even supersedes MtL in some cases (Table 4). Algorithms such as DT, SVM, and RLR, while presenting low residuals (Figure 4, Table 4), often produce reconstructed landmarks that present a number of deformities in certain isolated landmarks (Figure 5). This is potentially a product of the algorithms' inability to predict the values for multiple coordinates simultaneously, therefore losing the spatial—and consequently, the geometric and structural—relationship between landmarks. For this reason, the best performing regressors for RE are either NNs, MtL, or standard LR.

**TABLE 4:** Leave one‐out cross‐validation reconstruction error in millimeters of the Reverse Engineering (RE) when using MtL in comparison with Thin Plate Spline (TPS) interpolation for the prediction of different portions of femora and humeri with varying numbers of landmarks (LM) to be estimated.

	*N*° LM	RE			TPS			Neanderthal	
		Lower CI	Central	Upper CI	Lower CI	Central	Upper CI	RE	TPS
Femur distal	17	2.22	5.36	11.56	4.04	7.80	15.88	8.46	10.89
Femur proximal	19	2.67	5.52	11.77	6.65	13.25	27.03	9.67	11.33
Femur prox and dist	36	2.46	5.64	12.03	6.26	12.04	24.88	8.93	10.59
Femur diaphysis	164	2.18	4.16	7.60	3.73	6.86	13.72	3.69	5.28
Femur dist and diaphysis	181	3.08	8.50	19.90	14.39	34.22	60.32	4.98	11.15
Femur prox and diaph	183	3.25	7.52	17.70	9.30	30.35	63.08	7.80	12.28
Humerus distal	17 (17)	2.26	5.59	14.53	1.01	2.02	3.41	5.79	4.48
Humerus proximal	15 (13)	2.52	5.56	13.19	1.00	2.05	3.37	5.78	7.41
Humerus prox and dist	32 (30)	2.25	5.66	13.51	1.61	2.94	4.41	5.89	5.93
Humerus diaphysis	170 (170)	2.52	4.46	7.98	3.03	5.59	9.24	3.60	3.64
Humerus dist and diaph	187 (187)	3.02	7.32	16.30	8.33	27.12	57.60	13.45	12.83
Humerus prox and diaph	183 (181)	2.98	7.04	16.79	7.08	25.44	51.29	4.05	3.66

*Note:* Confidence intervals are calculated as 95% interquantile ranges. Central tendencies are reported as the median. Finally, the reconstruction error of the Neanderthal individuals has also been included. The number of missing landmarks appearing in brackets refers to the number of landmarks being predicted for the Neanderthal humerus, considering that 2 fixed landmarks are missing from the original individual.

In comparison with traditional approaches, such as TPS, the RE often works best, especially when large portions of the bone are missing. Both approaches exhibit their highest error rates when both the distal portion and the shaft are missing; however, the TPS approach shows substantially greater errors under these conditions (Figure 4, Table 4). For smaller portions of bone, however, the TPS method can sometimes be seen to be much more accurate, namely in our samples of humeri. Although the RE approach consistently achieves the lowest error rates in both the humerus and the femur when the shaft is missing, the TPS approach performs best in the humerus when only the distal portion is absent (Figure 4, Table 4).

**FIGURE 6:**
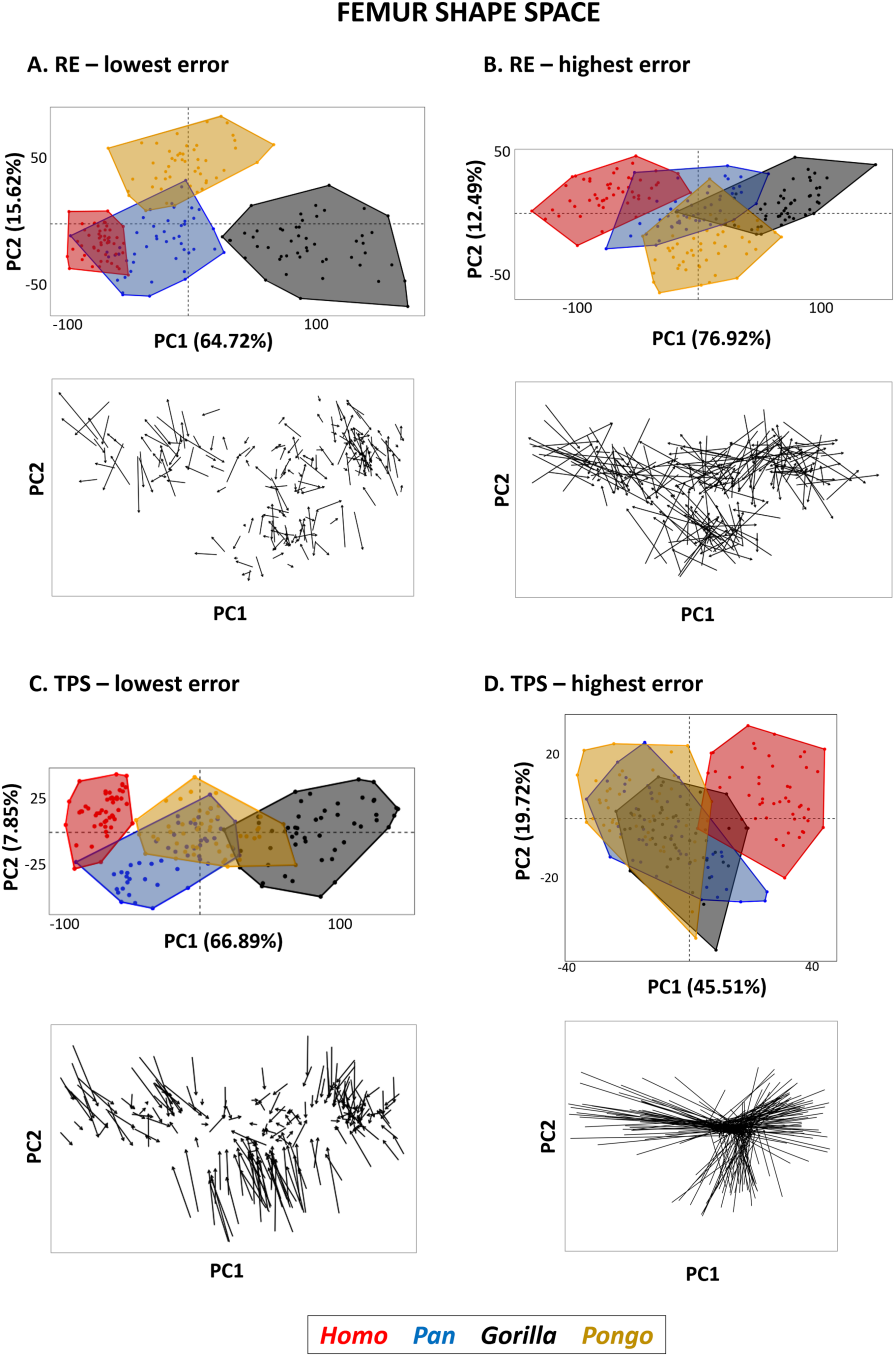
PCA plots in shape space for the femur reconstructions with the lowest (A and C) and highest errors (B and D) as defined in Table 4, computed using the MtL variant of the Reverse Engineering (RE) and Thin Plate Spline (TPS) approaches. The arrow plots illustrate the shift in position from the original PCA distribution (see Figures S3 and S4) to the feature space generated by the reconstructions.

**FIGURE 7:**
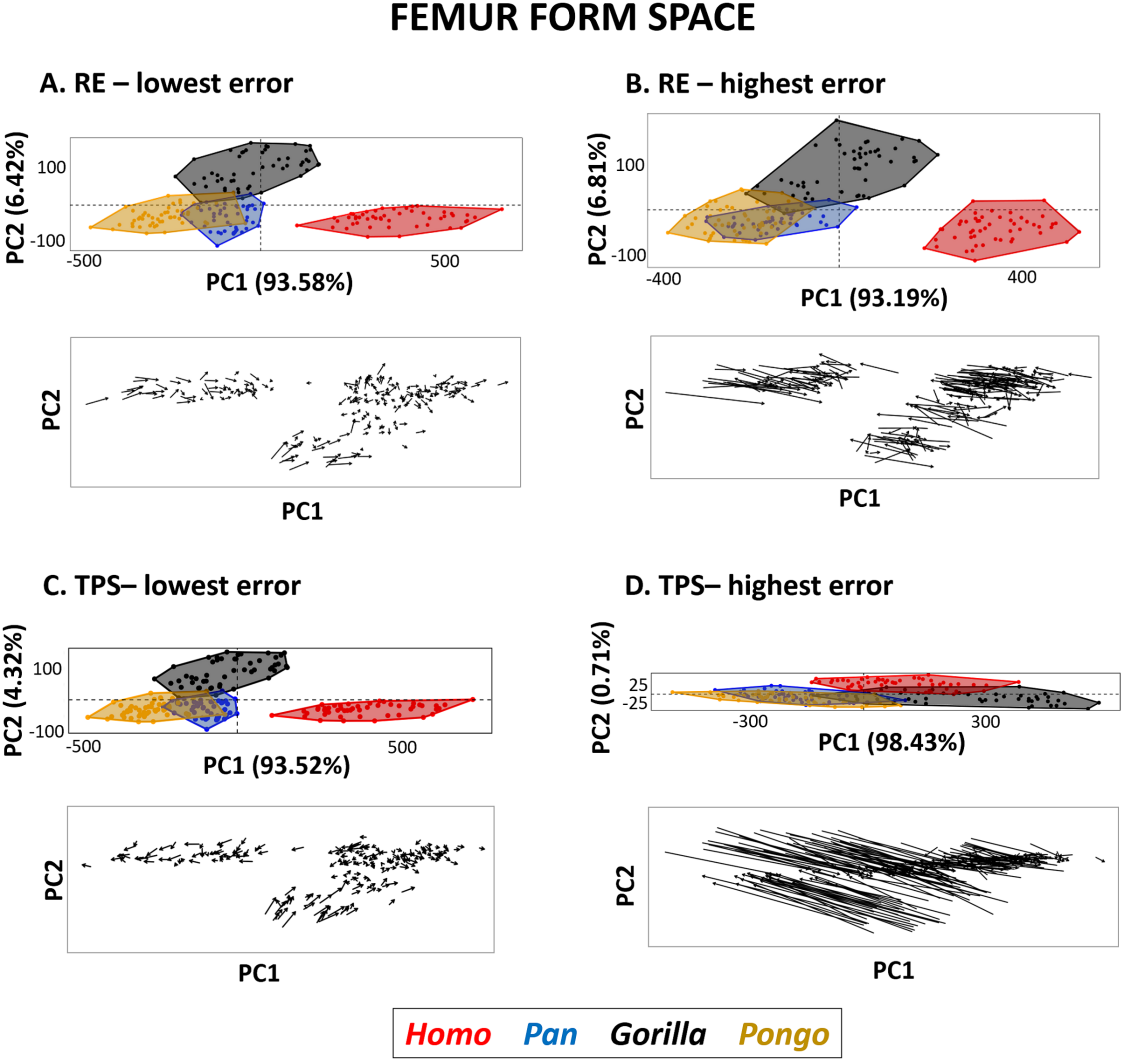
PCA plots in form space for the femur reconstructions with the lowest (A and C) and highest (B and D) errors as defined in Table 4, computed using the MtL variant of the Reverse Engineering (RE) and Thin Plate Spline (TPS) approaches. The arrow plots illustrate the shift in position from the original PCA distribution (see Figures S3 and S4) to the feature space generated by the reconstructions.

**FIGURE 8:**
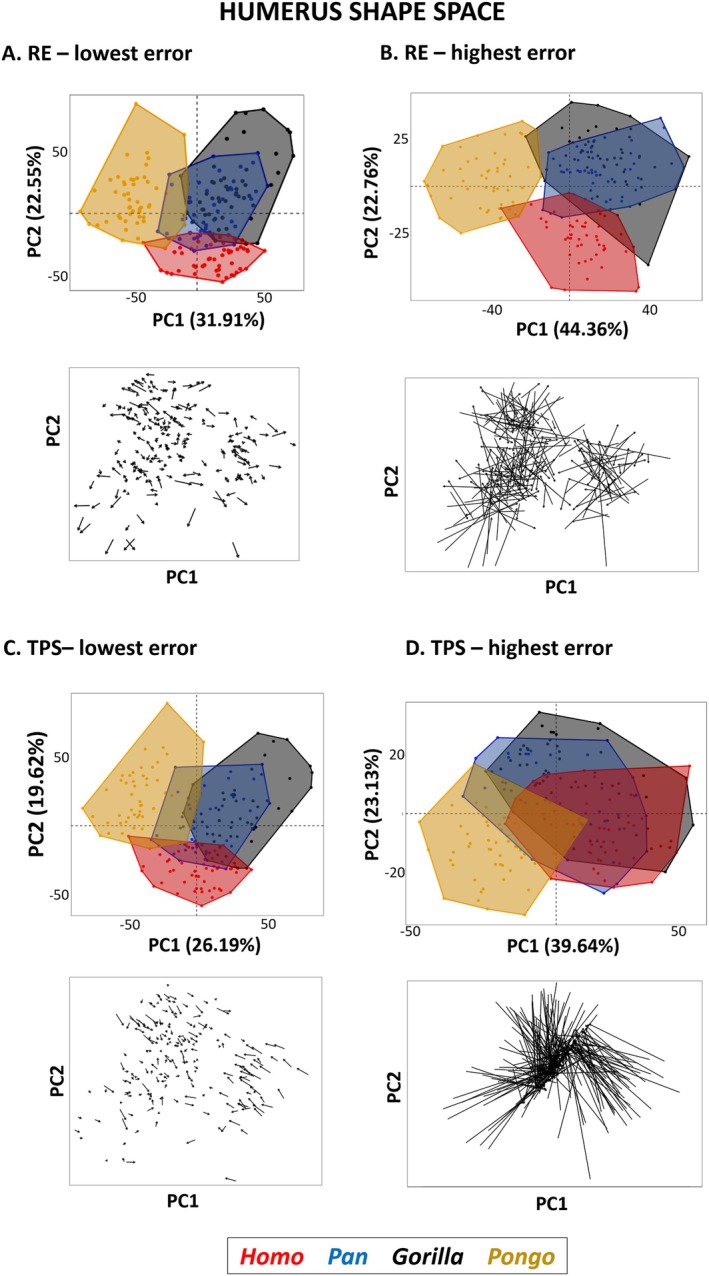
PCA plots in shape space for the humerus reconstructions with the lowest (A and C) and highest errors (B and D) as defined in Table 4, computed using the MtL variant of the Reverse Engineering (RE) and Thin Plate Spline (TPS) approaches. The arrow plots illustrate the shift in position from the original PCA distribution (see Figures S3 and S4) to the feature space generated by the reconstructions.

**FIGURE 9:**
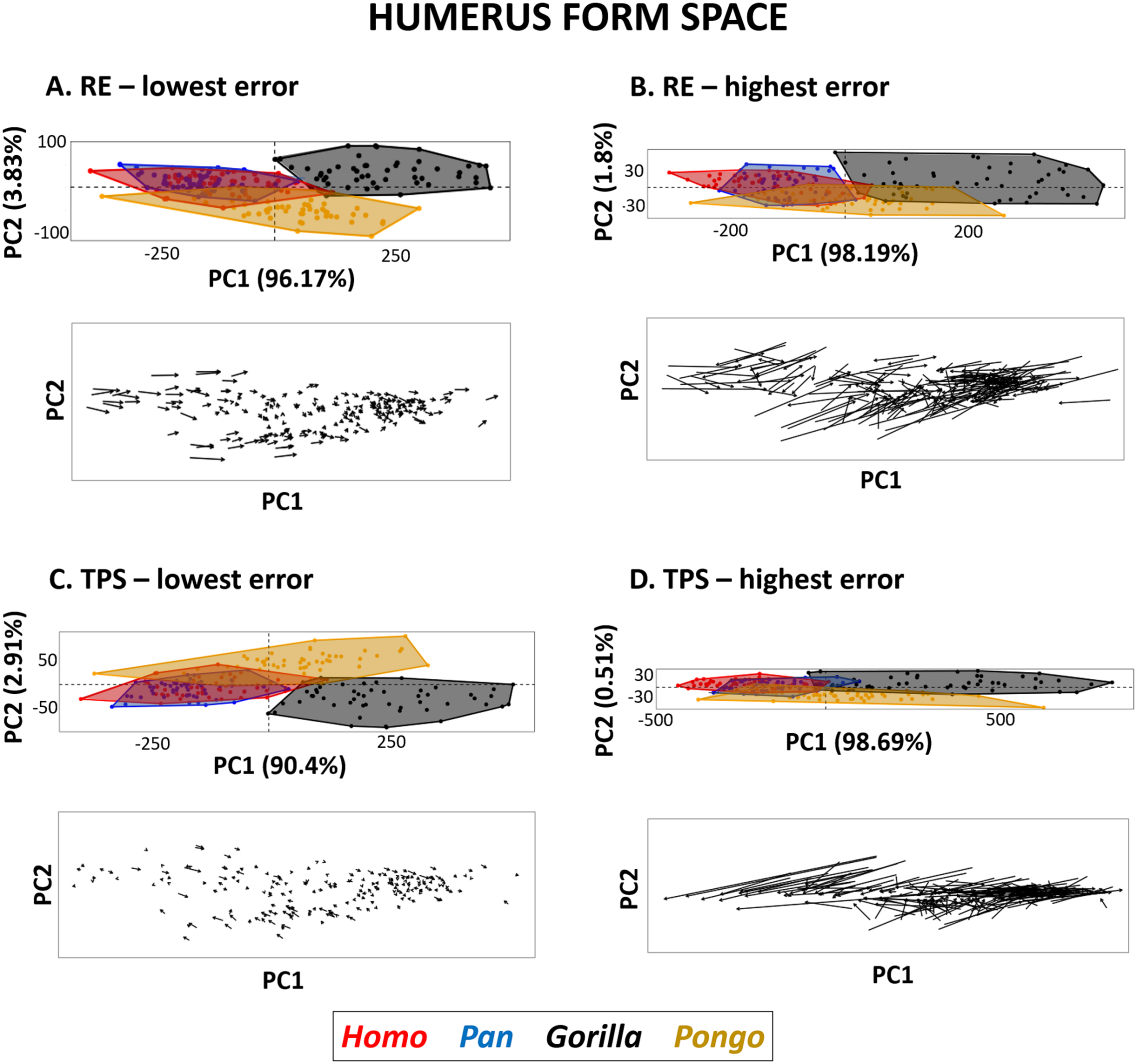
PCA plots in form space for the humerus reconstructions with the lowest (A and C) and highest errors (B and D) as defined in Table 4, computed using the MtL variant of the Reverse Engineering (RE) and Thin Plate Spline (TPS) approaches. The arrow plots illustrate the shift in position from the original PCA distribution (see Figures S3 and S4) to the feature space generated by the reconstructions.

When assessing the displacement of individuals in those PCA feature spaces generated using the missing landmark that produced the highest and lowest error rates (Table 4; Figures 6, 7, 8, 9), it can be seen that both TPS and RE approaches can cause some individuals to substantially shift position from the original distribution (Figures S3 and S4) when large portions of bone are missing. Nevertheless, it is fundamental to observe that in the case of RE, the biological signal is still maintained, while TPS‐based approaches can cause individuals to completely shift into regions of feature space that are occupied by different species. This is primarily observable when considering shape space (Figures 6 and 8), as the strong allometric patterns in form space ensure that the current sample of extant great apes remain separate (Figures 7 and 9). It is interesting to note that TPS is able to preserve to some extent this allometric component; long arrows across PC1 in Figures 7 and 9 are primarily due to the inverse flipping of PC1; however, the relationship between species generally remains the same. The largest important differences in these figures are therefore described by the change in PC2, where the magnitude of these changes reflects the loss of biological signal that is not as dependent on shape‐size relationships. Likewise, the scale of the PC2 largely changes, with a much smaller range in values across this dimension.

### 4.2 Reconstructing Neanderthal Individuals

Because the MtL variant of RE was found to perform best in our reference sample, all of the following results using RE therefore are performed using Reptile trained NNs.

**FIGURE 10:**
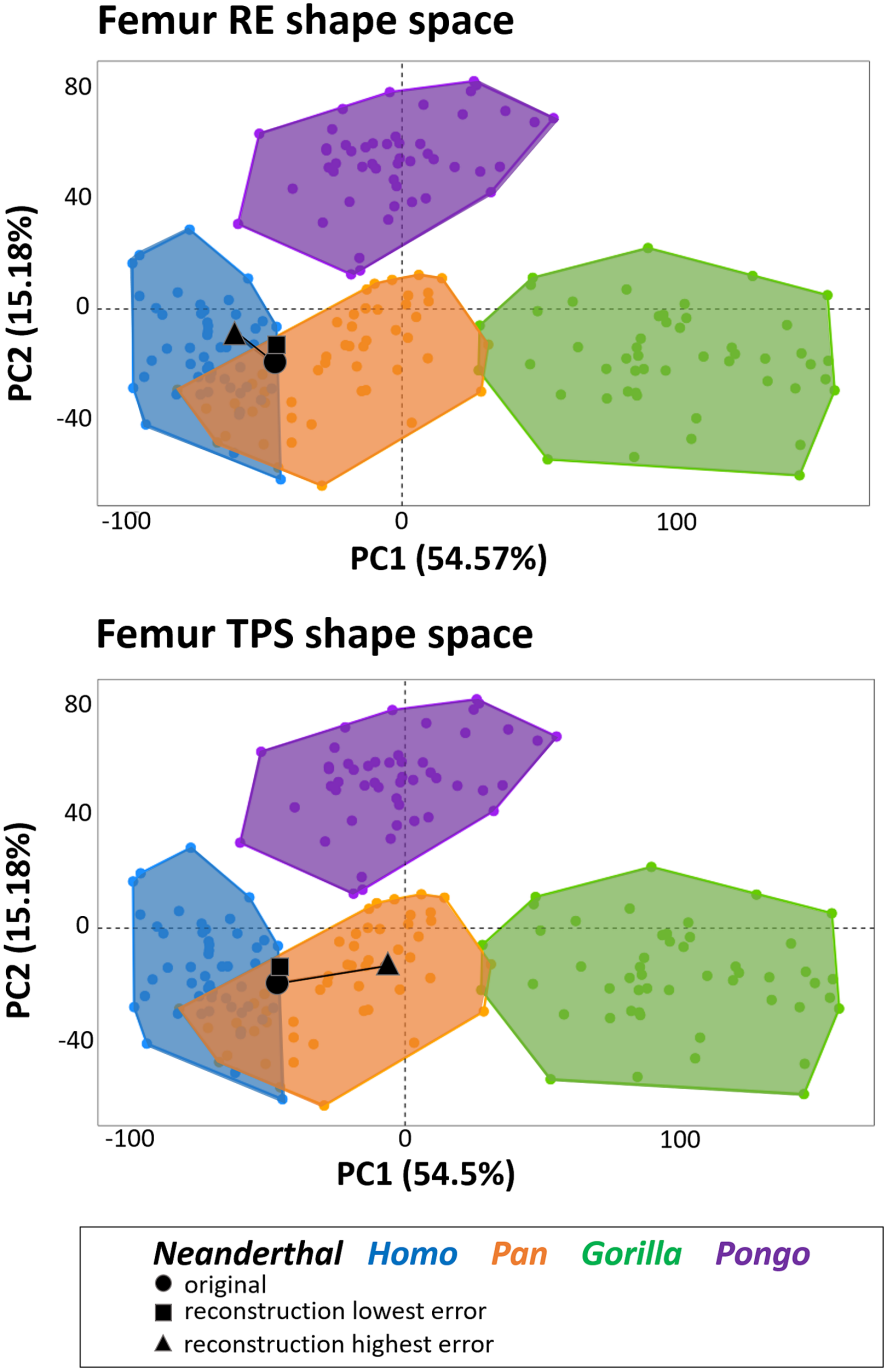
PCA plots in shape space, showing the original Neanderthal 1, along with the reconstructions by both the Reverse Engineering (RE), using MtL, and Thin Plate Spline (TPS) approaches, considering the lowest computed error and the highest computed error.

**FIGURE 11:**
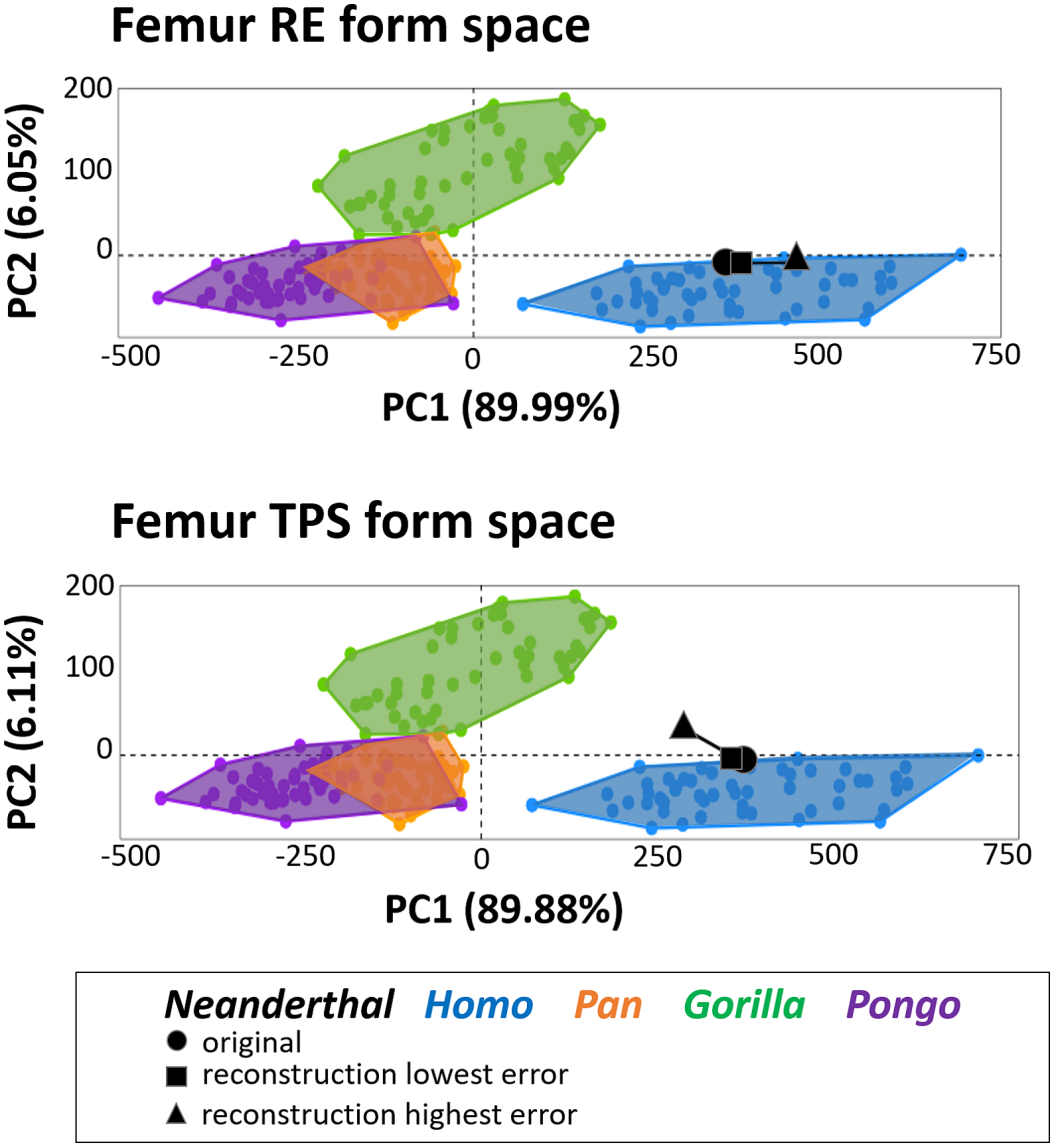
PCA plots in form space, showing the original Neanderthal 1, along with the reconstructions by both the Reverse Engineering (RE), using MtL, and Thin Plate Spline (TPS) approaches, considering the lowest computed error and the highest computed error.

**FIGURE 12:**
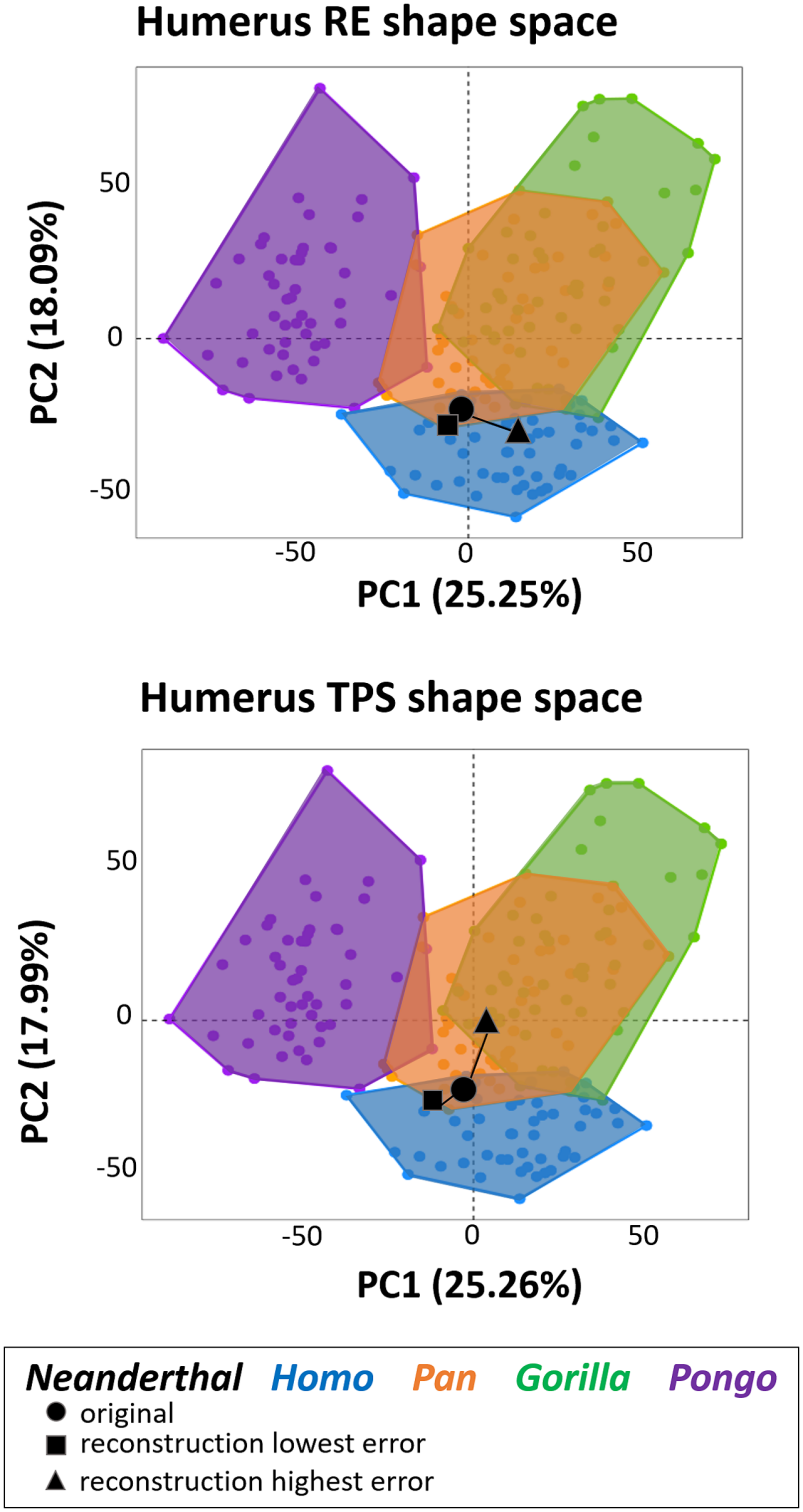
PCA plots in shape space, showing the original Simanya SI‐1, along with the reconstructions by both the Reverse Engineering (RE, using MtL) and Thin Plate Spline (TPS) approaches, considering the lowest computed error and the highest computed error.

**FIGURE 13:**
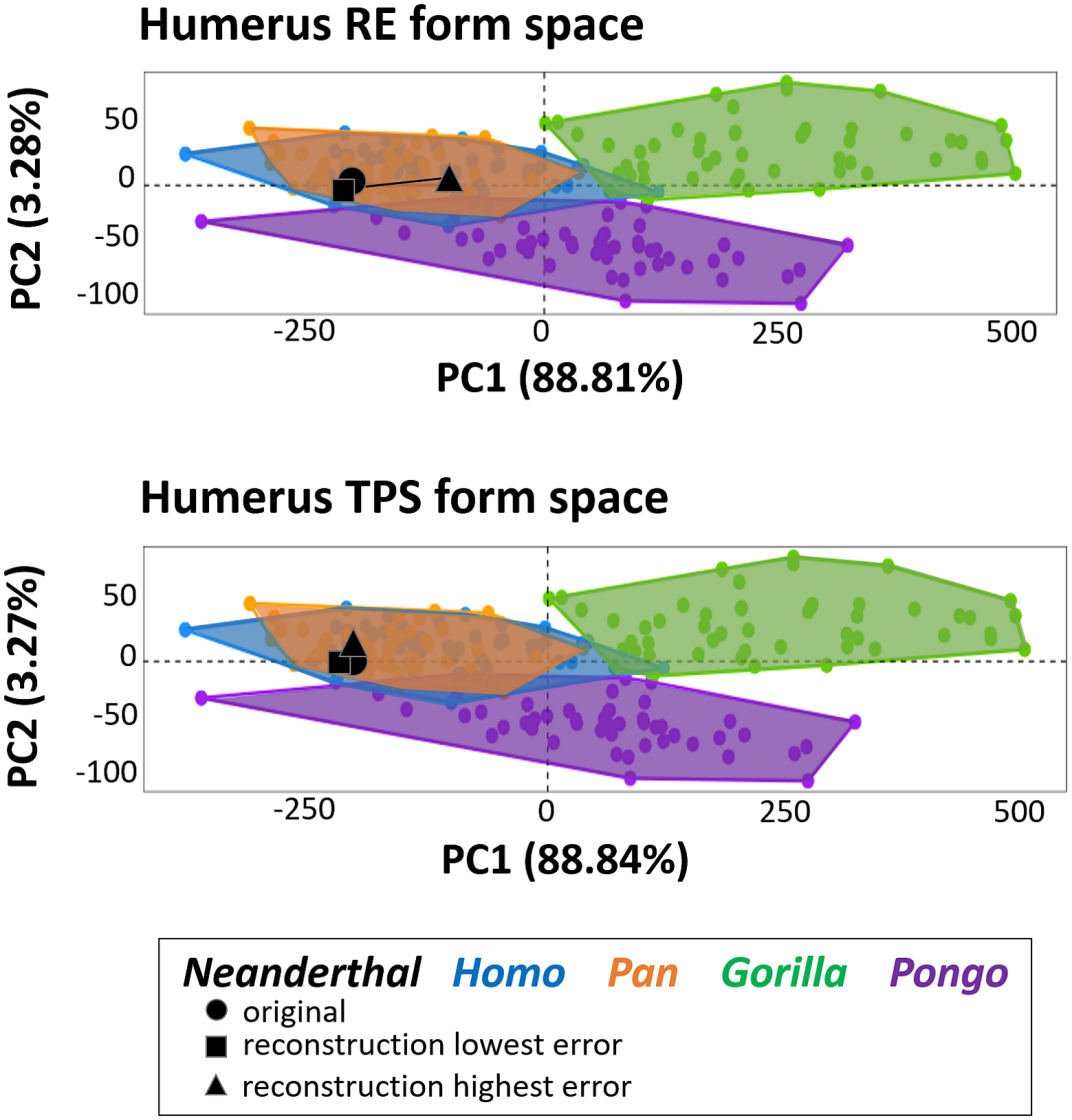
PCA plots in shape space, showing the original Simanya SI‐1, along with the reconstructions by both the Reverse Engineering (RE, using MtL) and Thin Plate Spline (TPS) approaches, considering the lowest computed error and the highest computed error.

The RE approach was able to efficiently reconstruct both Neanderthal individuals, regardless of the number of missing landmarks presented (Table 4). As expected, given the prior simulations, residuals increase as the percentage of missing landmarks increases; however, RE is able to produce relatively realistic reconstructions that, more importantly, do not place the individual in a position of feature space that would drastically change our interpretation of the specimen (Figures 10, 11, 12, 13).

According to our results, displacements in shape (Figures 10 and 12) and form (Figures 11 and 13) space are small when only a small portion of the fossil bone is reconstructed using both the RE and TPS methods, with displacements in form space being even more subtle. However, when the missing portion is larger, as shown in Figures 11 and 13, the displacement becomes more pronounced.

Interestingly, with the RE method, the Neanderthal femur consistently falls within the range described by humans in both shape and form space (Figures 10 and 11), closely mimicking the original position of the specimen along the first two PCs. In contrast, with the TPS method, the fossil specimen overlaps with *Pan* in shape space and falls outside the range of any extant great ape in form space (Figure 11).

A similar pattern is observed for the Neanderthal humerus (Figure 12), where the RE method preserves the relationship between the Neanderthal and modern humans, whereas the TPS method associates the specimen with other extant great apes. However, it is important to note that the reconstruction of the Simanya humerus in form space using the TPS method produces highly accurate results, with minimal positional shifts in form space, regardless of the size of the missing portion of the fossil bone (Figure 13).

Both the RE and TPS methods achieve the lowest errors when the diaphysis is reconstructed using information from both epiphyses (Table 4). In the femur, both methods encounter greater challenges reconstructing the proximal portion of the bone, whereas in the humerus, errors are more pronounced in the distal portion, particularly when both the diaphysis and the distal epiphysis need to be reconstructed. This significantly increases error compared to the reconstruction of other bone portions (Table 4).

**FIGURE 14:**
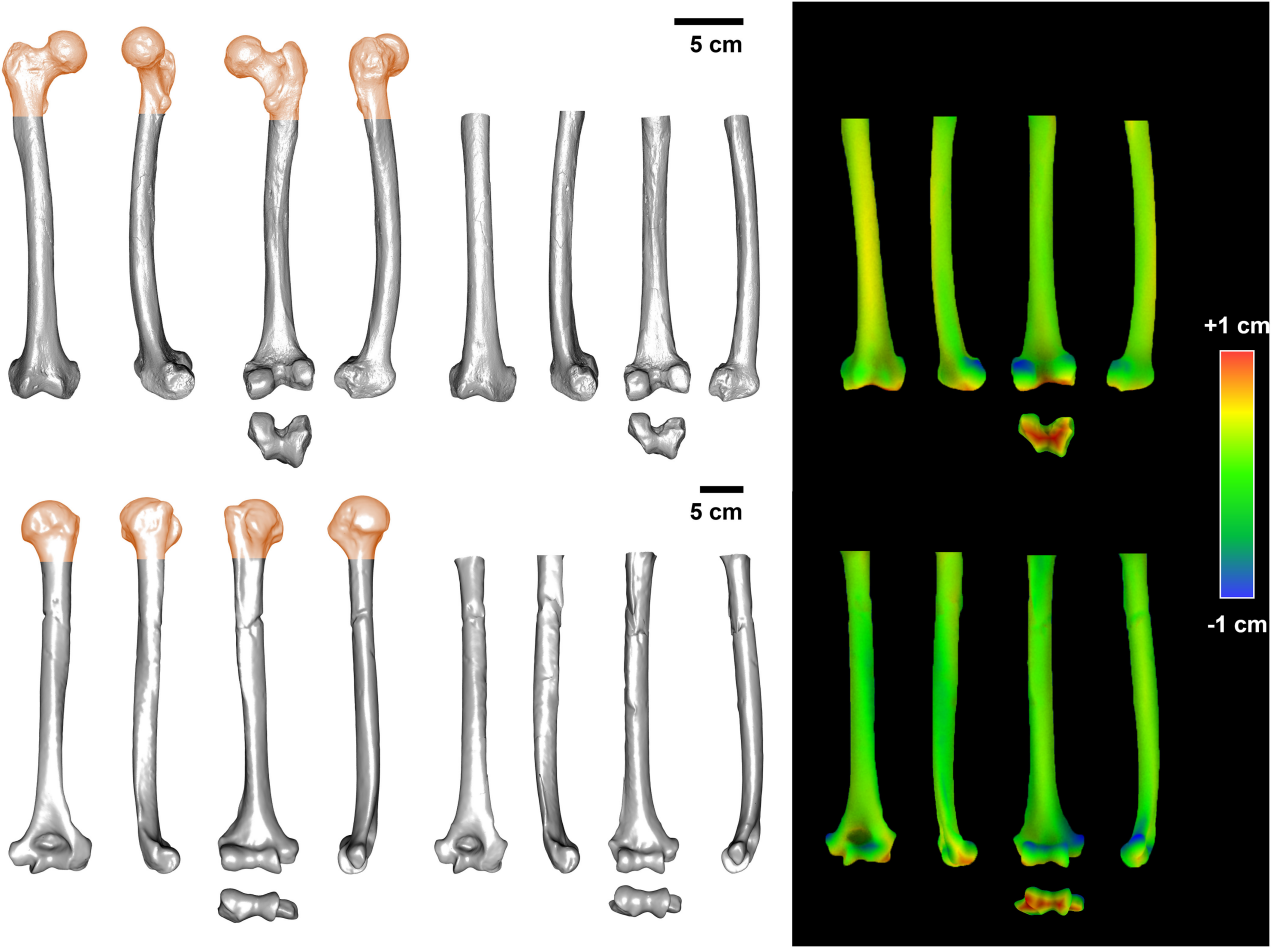
Visualization of the reconstructions of the distal epiphysis and diaphysis of the Neanderthal individuals using reptile based meta‐learning regression models. Left: The original femur and humerus. Center: The reconstructed femur and humerus. Right: Heatmaps representing the distance between the reconstructed mesh and the original mesh, with red indicating expansion, blue indicating contraction, and green representing no change.

When examining specific areas of error using the RE approach (Figure 14), the most distal portions of the Neanderthal femur and humerus appear expanded, potentially leading to a slight overestimation of bone length after reconstruction. Additionally, the medial epicondyle in the femur and the trochlea and capitulum in the humerus are reconstructed smaller than in the original individual. However, these errors remain highly localized. The rest of the distal epiphyses and the entire shaft show minimal differences between the original and reconstructed specimens, despite a large portion of the bones being reconstructed.

### 4.3 Recommendations for Real‐Life Applications

Several methodological considerations should be taken into account when applying the RE approach to empirical datasets.

First, reconstructed individuals generated in form space should not subsequently be subjected to GPA and analyzed in shape space using a reduced number of PCs. Because the RE approach already exploits covariance structure in the original feature space, projecting reconstructed specimens into a truncated PCA after GPA discards residual information that was integral to the reconstruction process. This loss of information can lead to distortions in the resulting feature space and artificially alter patterns of covariation and integration.

Second, the choice of the number of PC scores retained for reconstruction is a critical parameter that requires empirical tuning. Retaining too few PCs may oversimplify the underlying covariance structure, while retaining too many may introduce instability, particularly in high‐dimensional landmark datasets with limited sample sizes. Systematic experimentation is therefore required to identify an optimal balance between dimensionality reduction and information retention, depending on the anatomical element, sample size, and research question. Unfortunately, there is no golden rule for the optimal number of PCs that should be retained for any given analysis (Jollife 2002); therefore, the choice of PCs will be dependent on the analysts' questions and a detailed evaluation of precisely what methods are employed when using this approach.

Taken together, these considerations highlight the importance of maintaining methodological consistency between reconstruction and downstream analyses, as well as the need for careful evaluation of dimensionality choices when applying the RE approach to real‐world fossil material.

## 5 Discussion and Conclusions

At present palaeoanthropologists have at their disposal a relatively wide corpus of different methods for the reconstruction of incomplete fossils, a critical necessity in many fields of evolutionary and prehistoric research. Nevertheless, no single approach is universally applicable, and in many cases, a combination of methods is required to achieve a reliable reconstruction (Zollikofer et al. 2005; Gunz et al. 2011, 2012). This presents challenges when certain techniques are constrained by limitations, such as the absence of localized morphological data, or when comparative reference material is insufficient. Additionally, reconstructions often rely on comparisons with other individuals of the same species, ideally reflecting a shared ancestral pattern of variation rather than derived taxon‐specific variation. While this approach mitigates biases toward preconceived morphologies, it becomes problematic when fossil material is too fragmentary to allow even a broad taxonomic assignment.

One of the most widely used approaches for reconstructing missing landmarks is TPS interpolation. This method has proven particularly useful in reconstructing skull morphology, as it allows researchers to compare specimens with varying degrees of preservation (e.g., Gunz 2005; Gunz et al. 2009, 2011, 2012; Campione and Evans 2011). In more recent studies, approaches that explicitly exploit patterns of morphological covariance have been shown to be particularly effective and accurate for the reconstruction of missing anatomy (Torres‐Tamayo et al. 2025). The RE approach described here shares several underlying theoretical principles with such methods, most notably the assumption that morphological variation across different regions of a structure is not independent. In this sense, information captured in preserved areas of a bone can be informative of variation in regions that are missing.

Importantly, potential limitations in both the present approach and SSM do not primarily arise from the number of landmarks being predicted, but rather from the number of landmarks used to describe and parameterise the reference specimens themselves prior to models being fit. As the dimensionality of the reference shape representation increases, particularly through the inclusion of large numbers of landmarks or semilandmarks, the estimation of variance–covariance structure may become poorly conditioned (Marčenko and Pastur 1967; Donoho 2000; Aggarwal et al. 2001; Johnstone 2001; Ledoit and Wolf 2004; Bookstein 2017). High numbers of landmarks lead to instabilities in the covariance matrix on which both approaches rely heavily. This issue is exacerbated in fossil datasets, where sample sizes are necessarily limited and cannot readily compensate for high‐dimensional shape descriptions.

Moreover, both SSM and the present approach rely on covariance patterns that are inherently conditioned by the superimposition and alignment of landmark configurations. It has become increasingly apparent that morphological smearing (often referred to as the Pinocchio effect) and other factors can influence analyses of integration and covariation, stemming from the mathematical properties of Kendall's shape space and Procrustes superimposed coordinates (Bookstein 2016; Cardini 2019; Goswami et al. 2019; Klingenberg 2021; Zelditch and Swiderski 2023). While the present study employs a strict validation framework, particularly through careful implementation of leave‐one‐out cross‐validation, as detailed in Section 3.2 and File S2, to minimize artificially inflated reconstruction accuracies, the extent to which these effects influence reconstructions of fossil specimens remains difficult to predict. Addressing this limitation will require further targeted investigation. Nevertheless, throughout our analyses, it can be seen how the RE approach is still able to preserve morphological signal, highlighting the advantages of such an approach.

The present study introduces the RE approach as an additional tool in the morphometrician's repertoire for handling missing landmark data. Like all reconstruction methods, its effectiveness depends on the specific context and should be evaluated against alternative approaches. In some cases, a simpler method may yield comparable results, underscoring the importance of selecting the most appropriate tool for the task at hand. *Occam's razor* suggests that the simplest approach is often preferable, yet this is not always the case in morphological reconstructions, where complexity may be necessary to capture biological variation accurately.

Similarly, in data science, the “no free lunch theorem” states that no single algorithm performs best across all problems (Wolpert 1996; Wolpert and Macready 1997). This principle applies to fossil reconstruction, where no universal method can address all morphological gaps equally well. Future work should therefore focus on systematic evaluations of different approaches across various fossil datasets, assessing their relative strengths and limitations. By doing so, researchers can refine best practices for fossil reconstruction and enhance the reliability of morphological inferences in palaeoanthropology.

In the case of long bones, the RE approach has proven to be the best means of reconstructing very large portions of bone where symmetry cannot be exploited or leveraged for reconstructions. Long bones frequently appear as highly fragmentary remains in the fossil record, particularly in Pliocene and Early Pleistocene contexts. Combined with the scarcity of hominin fossils, this severely limits our understanding of morphological diversity in fossil groups, as it hinders direct comparisons among individuals. As a result, studies of long bones have often been restricted to specific bone portions (e.g., Almécija et al. 2013; Tallman 2012, 2013, 2014) or have included only a limited number of fossils due to difficulties in identifying homologous specimens (e.g., Aramendi et al. 2024; Arias‐Martorell et al. 2015).

Nevertheless, long bones are crucial for understanding locomotor adaptations, including key traits associated with humankind, such as tool use, or terrestrial bipedal walking, the origins of which have been the subject of intense debate, recently on the basis of femoral specimens (e.g., Cazenave et al. 2025; Daver et al. 2022). By integrating innovative tools encompassing techniques within virtual anatomies and deep and machine learning, the RE method has the potential to recover both morphological and biomechanical properties of fragmentary remains—for example by reconstructing features such as femoral neck length, femoral and humeral head proportions, epiphyseal orientation relative to the shaft, or even specific muscle attachment sites.

Additionally, by enabling the reconstruction of fragmented specimens that could not previously be analyzed together, the RE method could offer a more comprehensive perspective on postcranial diversity in the past. This is particularly meaningful in contexts with multiple penecontemporaneous species and complex taxonomic classifications (Grine et al. 2022; Antón and Middleton 2023). However, perhaps the most significant advantage of the RE method is its adaptability to other skeletal elements and taxa, making it useful for researchers in paleontology, anatomy, and biology—especially those working with incomplete bone elements and extinct species. In the latter case, the RE provides a valuable solution as the method is not as reliant on a single reference specimen or taxonomic group. By constructing mathematical functions using a sample that captures the widest possible range of morphological variability, the RE approach minimizes biases toward a single species, making it particularly valuable for the reconstruction of “unknown” morphologies like those represented by fossil remains. This is demonstrated by the arrow plots from PCA (Figures 6 and 7), where individuals do not shift as significantly within the morphospace as when reconstructions are performed using the alternative TPS approach. Likewise, when shifts occur, the morphological signal is preserved, preventing specimens from different species from being confused with one another, in contrast to results obtained using the TPS method.

Such results indicate that the RE method is less susceptible to agnostophobia—the challenge of predictive models handling data that differ radically from their training set (Dhamija et al. 2018). While agnostophobia remains a concern, it can be mitigated in morphological studies by selecting reference samples that encompass a broad spectrum of inter‐ and intraspecific variation, including sexual dimorphism, natural variability, and multiple species.

Nevertheless, it is likely that ontogenetic variation will still present a particular challenge, as significant shifts in shape and form trajectories that were not accounted for during training may impact predictions. In the present study, the use of form space revealed that when large portions of Neanderthal humeri and femora were missing, the total length of the reconstructed individuals tended to be smaller than the original specimens, resulting in a shift across PC1. Despite this, non‐size‐related features along PC2 remained stable, preserving the phylogenetic and morphofunctional signal. For this purpose, it is important to consider the variable size in reconstructions if allometric patterns are particularly notable. Therefore, we emphasize that the careful selection of a reference sample encompassing both shared and diverse patterns of variation in shape and form remains the most effective strategy for handling missing data, even when utilizing the RE approach.

In many cases, the calculated residuals remain higher than would be desirable for certain applications (Table 4 reports an average of 6 mm for the RE approach). For example, if tasks such as reintegrations or prosthetic design relied on these reconstructions, they might not achieve the necessary metrical precision. Nevertheless, for the purpose of the geometric morphometric analysis of fossil remains, the resulting reconstructions appear perfectly comparable with the referential datasets. Across all PCAs and corresponding assessments, it can be seen that the morphological signal—whether phylogenetic or biomechanical—remains intact while achieving greater precision compared to methods such as TPS in this specific case study. This effect is particularly evident in the observed shifts in taxonomic affiliation among Neanderthal individuals in some analyses, who present greater affinities in morphospace to *Pan* when reconstructed using TPS protocols for femora, and also *Gorilla* for humeri. Such results would lead to drastically different interpretations of the results, which could be considered particularly problematic for many case studies.

In conclusion, the RE approach offers a promising addition to the available methods for fossil reconstruction by reducing a number of biases that have, in the past, proved particularly problematic. While it is not a universal solution, its ability to retain phylogenetic and biomechanical signals while improving precision over TPS highlights its potential for palaeoanthropological applications. Nevertheless, challenges are still present that require further investigation, highlighting the importance of more comprehensive referential datasets that capture true inter and intra‐specific variability as much as possible. Future research should further refine the RE approach and assess its performance on a number of different applications and types of bones. By advancing reconstruction methodologies, we can improve our understanding of evolutionary processes leading to more rigorous and reproducible approaches in the study of extinct species.

## Author Contributions

**Lloyd A. Courtenay:** conceptualization, methodology, software, validation, formal analysis, visualization, writing – original draft, writing – review and editing, investigation. **Julia Aramendi:** validation, data curation, formal analysis, investigation, resources, writing – original draft, writing – review and editing, project administration, funding acquisition.

## Funding

L.A.C. is funded by the Agence Nationale de la Recherche, for the Access‐ERC project BSMART (ANR‐25‐AERC‐0005). J.A. was supported by the British Academy Newton International Fellowship during the data collection process (Grant number: NIF22\220310), and is currently funded by the Centre National de la Recherche Scientifique (CPJ‐Hominines) and the Agence Nationale de la Recherche (ANR‐24‐CE02‐2903).

## Conflicts of Interest

The authors declare no conflicts of interest.

## Supporting information


**Figure S1:** Anatomical landmarks used to describe the humeri and femora on a *Pongo* specimen.
**Figure S2:** Templates and targets used during the sliding process. (A) Simplified cylindrical template; (B) Consensus templates; (C) Results after semilandmark projection and sliding in the four modern great ape groups.
**Figure S3:** Original PCA plots in shape and form space for the modern great ape femora.
**Figure S4:** Original PCA plots in shape and form space for the modern great ape humeri.

**File S2:** Code for the evaluation and execution of the Reverse Engineering.

## Data Availability

Fossil data analyzed in this study derive from previously published specimens for which redistribution is restricted by the original data providers. Access to these specimens should be requested directly from the original authors cited in this study. Landmarks for extant comparative specimens are available from https://doi.org/10.5281/zenodo.20677163. File S2 provides sample code for the replication of methods.
