# Supplementary material for: Missing Landmark Estimation Using Reverse Engineering: Challenges and Potential Solutions for the Study of Hominin Long Bones
Source: Am J Biol Anthropol. 2026 Aug 3;190(4):e70334. doi: 10.1002/ajpa.70334 (PMC13430587; doi:10.1002/ajpa.70334)
Supplement: Supplementary file 1 — Figure S1: Anatomical landmarks used to describe the humeri and femora on a Pongo specimen. Figure S2: Templates and targets used during the sliding process. (A) Simplified cylindrical template; (B) Consensus templates; (C) Results after semilandmark projection and sliding in the four modern great ape groups. Figure S3: Original PCA plots in shape and form space for the modern great ape femora. Figure S4: Original PCA plots in shape and form space for the modern great ape humeri. [file AJPA-190-e70334-s001.pdf]

Missing Landmark Estimation using Reverse Engineering; challenges and potential solutions for the study of hominin long bones

Supplementary File 1

Lloyd A. Courtenay [1, 2], Julia Aramendi [3]

[1] CNRS, PACEA UMR5199, Université de Bordeaux, Bât B2, Allée Geoffroy Saint Hilaire, CS50023, Pessac 33600, France
[2] Department d'Història I Història de l'Art, Universitat Rovira i Virgili, Avinguda de Catalunya 35, 43002, Tarragona, Spain
[3] Laboratoire de Paléontologie, Évolution, Paléoécosystèmes et Paléoprimatologie (PALEVOPRIM), UMR 7262, CNRS & Université de Poitiers, Poitiers, France

Corresponding author: Lloyd Austin Courtenay
Email: ladc1995@gmail.com
OrcID: 0000-0002-4810-2001

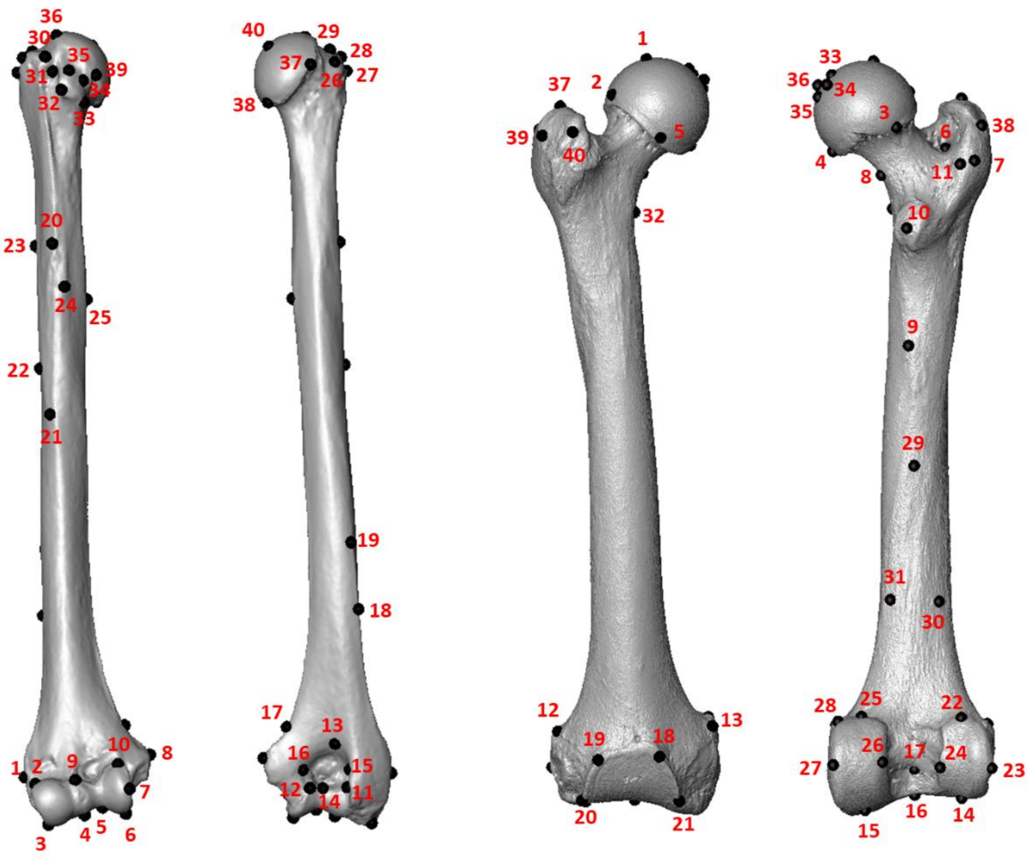

Figure S1. Anatomical landmarks used to describe the humeri and femora on a *Pongo* specimen.

To make the projection and sliding of semilandmarks automatic, a cylindrical template was designed to represent an augmented and simplified version of the long bone shaft (Figure S2). A total of 160 points were distributed in 20 equally spaced section lines along the cylinder, with each horizontal section containing 8 points homogeneously distributed. The distance between points was calculated to ensure equidistance, and the points were located on the cylinder

surface using Avizo. To project the point cloud on each bone and constrain the sliding process of the semilandmarks, thus maintaining geometric homology among specimens, eight of the 160 points were treated as fixed points. These eight fixed points were alternately located in the upper and lower sections. Consequently, each of these two sections combined four semilandmarks with four fixed landmarks. The remaining 152 points were shaped as surface semilandmarks.

The location of the reference fixed points met two main criteria: first, the need to fulfill the homology requirement among specimens, and second, the intent to map as much diaphyseal surface as possible. To achieve this, anatomical landmarks were carefully investigated throughout the entire sample, with a focus on establishing an upper and an equivalent lower point that could be easily identified among all specimens. The arrangement of these two primary points allowed for the location of the rest of the fixed points, taking into account the rule of equidistance. Notable anatomical structures that were easily identifiable along the longitudinal axis of all great ape long bones were chosen for each skeletal element to describe the starting proximal and distal points. After careful examination, the medial crest of the humerus and the linea aspera of the femur were selected as anatomical references. To ensure maximal precision and homogeneity, cross-sectional cuts were taken on each long bone model individually in Avizo. Cuts were performed at heights established as homologous for each species and element, and fixed points were located equidistantly on the proximal and distal shaft areas.

After sliding the semilandmarks, a Generalized Procrustes Analysis (GPA) was performed to calculate the average shape of the humerus and femur. The consensus shape for each long bone was created by considering all the slid specimens and scaling them to the mean centroid size. The calculated mean coordinates were used to generate a mean surface for each skeletal element. The surfaces of a chimpanzee humerus and femur were used to warp two mean surfaces based on the mean coordinates extracted from the GPA analyses. The chimpanzee surface was preferred because it is considered the best proxy for the average shape among the four modern primate species used in this study. The use of an average or mean shape as a reference is often mentioned in the literature (Gunz and Mitteroecker, 2013; Mitteroecker and Gunz, 2009) and serves to optimize semilandmark projection and sliding by minimizing shape differences between the template and the targets. Sliding was performed iteratively to reduce bending energy as a criterion for establishing homology. The calculated means were then used as templates to perform a second round of sliding on the modern comparative sample (Figure S2). The landmarking and semilandmark projection and sliding were performed using the EVAN Toolbox.

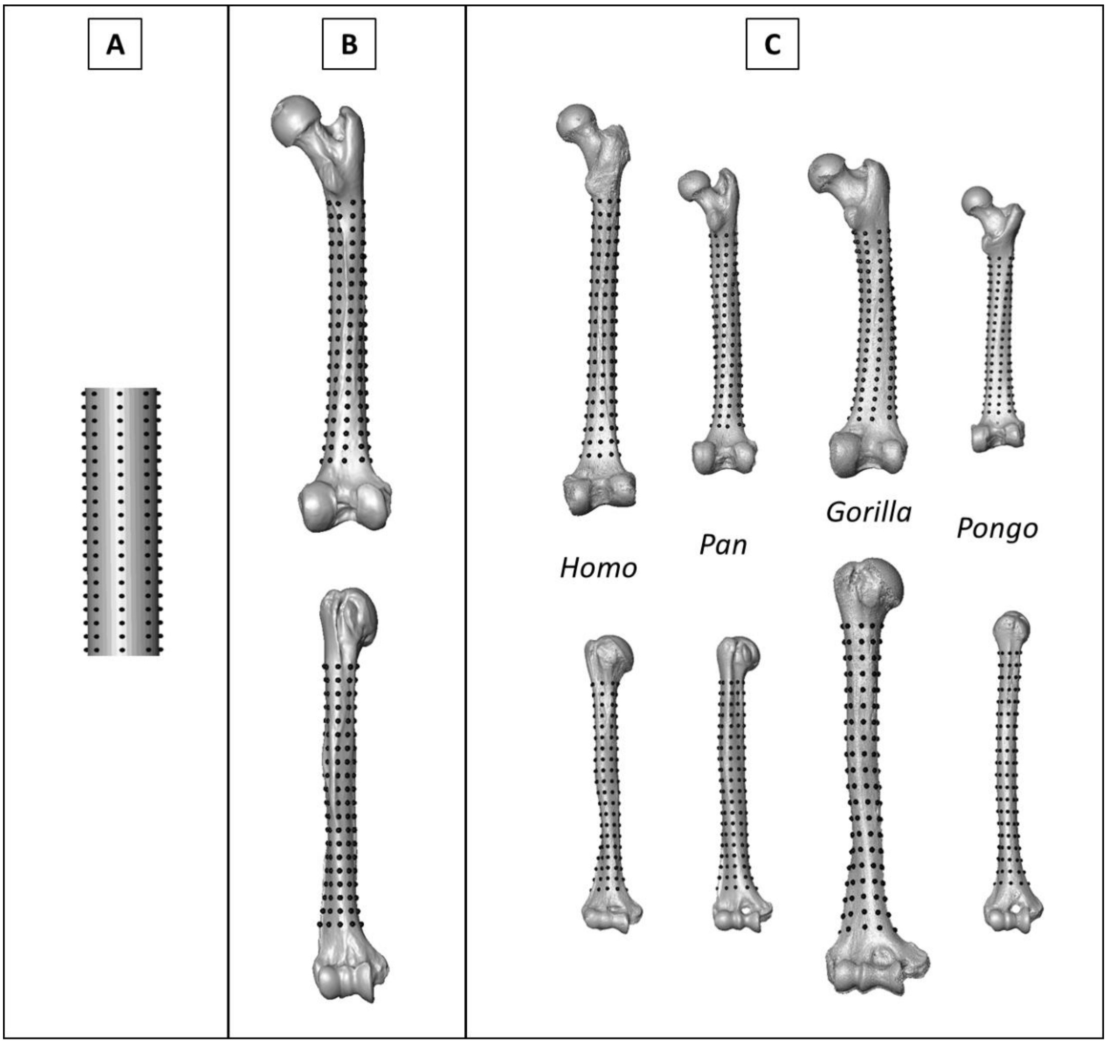

Figure S2. Templates and targets used during the sliding process. A) Simplified cylindrical template; B) Consensus templates; C) Results after semilandmark projection and sliding in the four modern great ape groups.

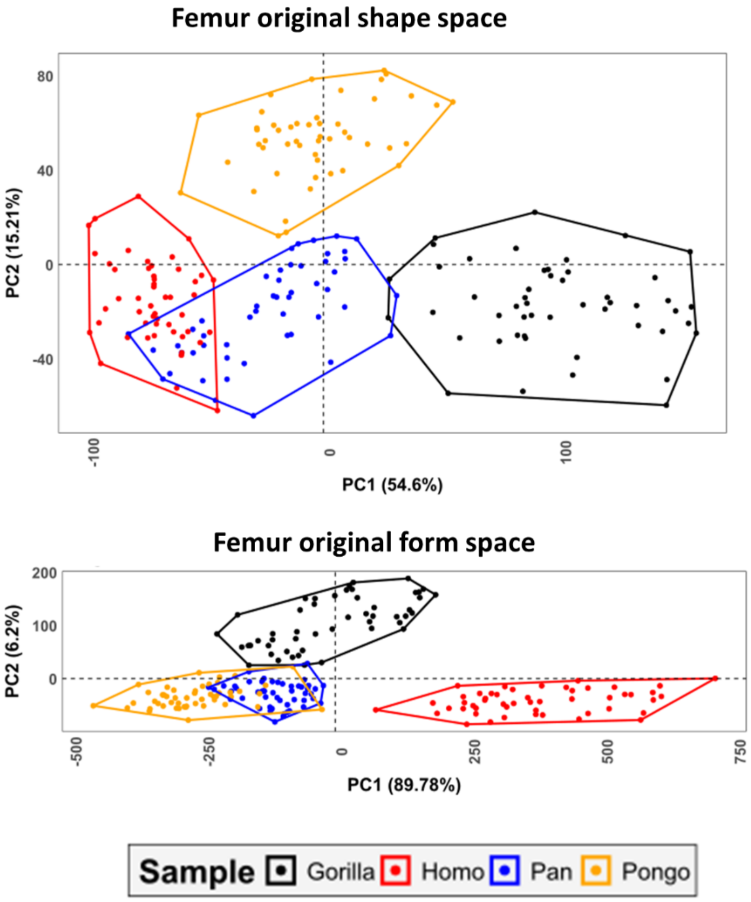

Figure S3. Original PCA plots in shape and form space for the modern great ape femora.

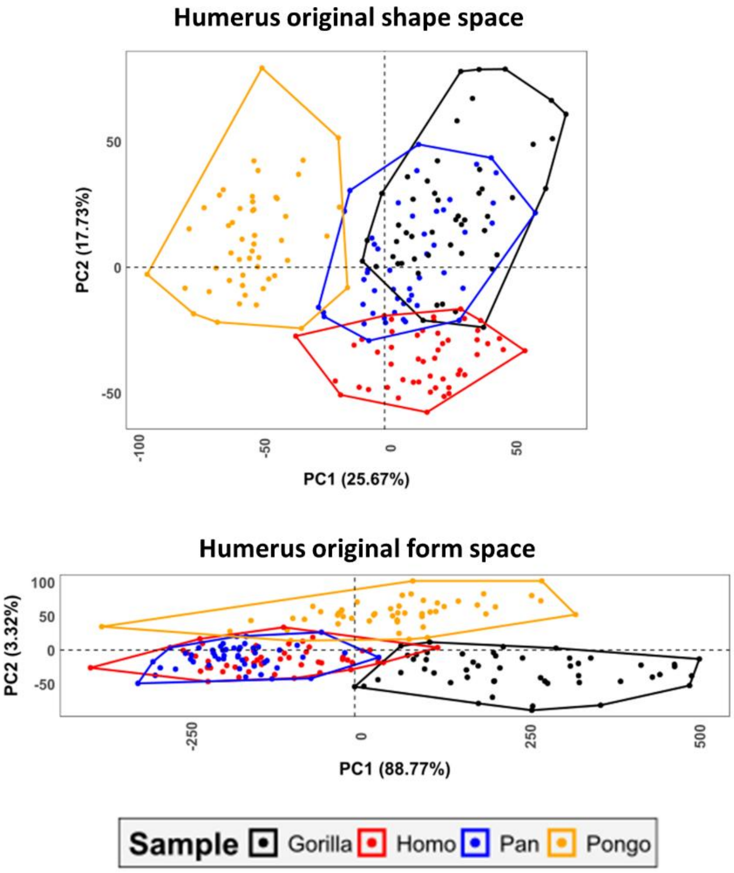

Figure S4. Original PCA plots in shape and form space for the modern great ape humeri.

References

Gunz, P., Mitteroecker, P., 2013. Semilandmarks: a method for quantifying curves and surfaces. Hystrix, the Italian Journal of Mammalogy 24, 103-109.

Mitteroecker, P., Gunz, P., 2009 Advances in Geometric Morphometrics. *Evolutionary Biology* 36, 235-247
