# Supplementary material for: Missing Landmark Estimation Using Reverse Engineering: Challenges and Potential Solutions for the Study of Hominin Long Bones
Source: Am J Biol Anthropol. 2026 Aug 3;190(4):e70334. doi: 10.1002/ajpa.70334 (PMC13430587; doi:10.1002/ajpa.70334)
Supplement: Supplementary file 2 — File S2: Code for the evaluation and execution of the Reverse Engineering. [file AJPA-190-e70334-s002.pdf]

Missing Landmark Estimation using Reverse Engineering; challenges and potential solutions for the study of hominin long bones.

Supplementary File 2: Code for the evaluation and execution of the Reverse Engineering approach

Lloyd A. Courtenay[1,2,*] **and Julia Aramendi**[3]

[1]CNRS, PACEA UMR5199, Université de Bordeaux, Pessac, France
[2]Department d'Historia i Historia de l'Art, Universitat Rovira i Virgili (URV), Tarragona, Spain
[3]CNRS, PALEVOPRIM UMR7262, Université de Poitiers, Poitiers, France
[*]Corresponding Author Email: ladc1995@gmail.com

The present document contains a brief documentation of the protocol and algorithm for evaluating the reverse engineering approach, as well as the necessary code to train a meta-learning regression algorithm in Python

Contents

Computer Setup 2
Evaluation Workflow . . . 2
R Implementation 3
Dependencies . . . 3
Utility Functions . . . 3
Data Input and Preprocessing . . . 4
Linear Regression Model . . . 4
Evaluation . . . 6
Alternative Regression Models . . . 6
Potentially Cleaner Implementation . . . 7
Python Implementation 12
Dependencies . . . 13
Metalearning Evaluation Code . . . 13
Potentially Cleaner Implementation . . . 16

Computer Setup

Two different programming languages are used for the different aspects of this paper. The majority of applications use the R programming language, which require a number of libraries to be installed. The meta-learning portion of this methodology, however, is conducted using the Python programming language. All code was run on a Windows operating system.

Before we continue, two R libraries have to be installed that cannot be installed from the basic CRAN. The first library is **GraphGMM** and the second **pValueRobust**, both of which are available as GitHub repositories in the corresponding author's GitHub page.

To install an R library from a GitHub repository the user must first have installed **RTools**, and then the library **devtools**. For those unfamiliar with this package, the **devtools** package is a set of tools used for the development and management of R packages. **devtools** will not work properly, however, if **RTools** is not installed first and added to the system path. Most recent versions of **RTools** already add **RTools** to the path, older versions need this to be done manually.

devtools can simply be installed using;

```
install.packages("devtools")
```

Once **devtools** has been installed, we can use the *install_github* function to access the **GraphGMM** and **pValueRobust** repositories and install the library.

```
library(devtools)
install_github("LACourtenay/GraphGMM")
install_github("LACourtenay/pValueRobust")
```

For the Python portion of this study, it is highly recommended that the user installs Python using **Anaconda**, and sets up a Conda environment specifically for this study. The user *has* to install **TensorFlow** for the Python version of this code. It is important to note however that **Tensorflow** is a very specific library, and can be frustrating to use when updates occur. When the library is updated, many functions begin to work differently, may have slightly different names, or may present problems, when they didn't before. It is **VERY** important that you use specifically the versions of libraries presented in this document. We recommend that you do this by having an Anaconda environment specifically established for this project with those precise library and package versions. Please do not blindly run the code on any computer thinking that the code will work without checking that your computer meets these requirements.

Evaluation workflow

This section summarises the evaluation procedure used to assess landmark reconstruction accuracy. The workflow is presented algorithmically for clarity and corresponds directly to the R and Python implementations described in the following sections.

Algorithm 1: Leave-one-out evaluation workflow for landmark reconstruction

Input : Dataset (D) of n specimens
Output : Measurement Error

for $i \leftarrow 1$ **to** n **do**
- Split D into $D \setminus S_{val}$ (training) and S_{val} (validation), where $val = i$;
- Compute GPA without scaling on $D \setminus S_{val}$ to get X;
- Define D' with missing landmarks LM' and perform GPA again to get X';
- Compute morphological descriptors z from X';
- Establish a mapping $f : z \rightarrow X$;
- Use $f(z)$ to predict the coordinates of LM' for S_{val}, defining S'_{val};
- Append S_{val} to S'_{val} and X, and compute GPA without scaling;
- Compute RMSE between the superimposed coordinates of S_{val} and S'_{val};

R Implementation

This section documents the R implementation used to predict missing landmark coordinates using linear regression models within a geometric morphometric framework. The code is intended to reproduce the analyses reported in the main text and is not presented as a general-purpose software package.

Dependencies

The following R packages are required (or recommended):

- **GraphGMM** (v.1.0.0)
- **pValueRobust** (v.1.0.0)
- **abind** (v.1.4-8)
- **geomorph** (v.4.0.9)
- **shapes** (v.1.2.7)
- **Morpho** (v.2.12.0)

For those interested in trying the different regression models described in the original paper, then these additional libraries are required:

- **rpart** (v.4.1.23)
- **e1071** (v.1.7-14)
- **caret** (v.6.0-94)
- **MASS** (v.7.3-60.2)

Utility Functions

```r


# Euclidean distance between two vectors

euclidean <- function(a, b) {
    return(sqrt(sum((a - b)^2)))
}

# Root Mean Square Error

rmse <- function(residuals) {

  residuals <- residuals ^ 2
  mse <- mean(residuals)

  return(sqrt(mse))

}

#
```

```


# Summary statistics for residual distributions

describe_residuals <- function(residuals, indx = NULL) {

  shapiro_p <- shapiro.test(residuals)$p.value
  ci <- quantile_CI(residuals)

  cat("\n")
  cat(paste0("Lower CI: ", ci[1], "\n"))

  if (shapiro_p < 0.003) {
    cat(paste0("Mean:  ", mean(residuals), "\n"))
  } else {
    cat(paste0("Median:  ", median(residuals), "\n"))
  }

  cat(paste0("Upper CI: ", ci[2], "\n\n"))

}

#
```

Data Input and Preprocessing

Landmark configurations are read from Morphologika (`.txt`) files and aligned using Generalized Procrustes Analysis (GPA) without scaling. A subset of landmarks corresponding to the preserved anatomical region is retained and used to derive morphological descriptors via principal component analysis (PCA). PCA scores are computed from vectorised landmark configurations.

```


# load landmark dataset

complete_specimens <- read.morphologika(
  ".\\path\\to\\landmark_data.txt"
)

# perform Generalised Procrustes Analysis (no scaling)

x <- GPA(complete_specimens$coords, scale = FALSE)


# select subset of landmarks to be retained or observed

landmark_indices <- c(26:38)


# perform GPA on the observed landmark subset

x_partial <- GPA(
  complete_specimens$coords[landmark_indices,,],
  scale = FALSE
)


# compute low-dimensional morphological descriptors

z <- pca_plot(
  vector_from_landmarks(x_partial$coordinates)
)$pc_scores

#
```

Linear Regression Model

Missing landmarks are predicted using a leave-one-out procedure. For each individual, a linear model is fitted between morphological descriptors (PCA scores) and full landmark configurations from the remaining specimens. The left-out individual

is reconstructed from its descriptors and aligned to the original sample using GPA prior to evaluation.

```r

# define the number of pc scores we wish to use for reconstructions

pc_scores <- 10

# initialize residuals vector

residuals <- c()

for (individual in 1:dim(dataset$coordinates)[3]) {

  # define target and training sets

  target_individual <- dataset$coords[,,individual]
  training_set <- dataset$coords[,,-individual]

  # GPA on training set

  gpa_training <- GPA(training_set, scale = FALSE)

  # GPA on subset of landmarks for descriptor extraction

  gpa_landmarks <- GPA(dataset$coords[landmark_indices,,], scale = FALSE)

  # compute morphological descriptors (PC scores)

  pc_scores <- pca_plot(vector_from_landmarks(gpa_landmarks$coordinates))$pc_scores[,1:pc_scores]

  # predict missing landmarks for target individual

  predicted_morphology <- morphology_predictor(
    gpa_training$coordinates,
    as.matrix(pc_scores[-individual,]),
    as.matrix(pc_scores[individual,])
  )

  # align original and predicted coordinates via GPA

  aligned <- GPA(
    abind(dataset$coords, predicted_morphology, along = 3),
    scale = FALSE
  )

  original_individual <- aligned$coordinates[,,individual]
  reconstructed_individual <- aligned$coordinates[,,dim(gpa_landmarks$coordinates)[3] + 1]

  ind_residuals <- c()
  for (landmark in 1:dim(original_individual)[1]) {
    ind_residuals <- c(
      ind_residuals,
      euclidean(original_individual[landmark,], reconstructed_individual[landmark,])
    )
  }

  ind_rmse <- rmse(ind_residuals)
  residuals <- c(residuals, ind_rmse)

}

#
```

Note: The *morphology_predictor* function provided by the **GraphGMM** library is simply our own implementation of the *shape.predictor* function from **geomorph**. In fact, this line can easily be substituted by the functions from **geomorph** if the user does not want to use other libraries.

Evaluation

Reconstruction accuracy is quantified using landmark-wise Euclidean distances between original and reconstructed configurations. Errors are summarised per individual using RMSE values. Confidence intervals are estimated using quantile-based robust statistics.

```

# summarise residuals

describe_residuals(residuals)

#
```

Alternative Regression Models

To implement other regression algorithms, line 31 has to be replaced with the following snippets of code.

Robust Linear Regression

Because the RL model does not produce multivariate outputs, the coordinates have to be predicted one by one. For this purpose we have to flatten the matrix, and then regress for each coordinate value separately.

```

flattened_x_prima <- vector_from_lanmdarks(x$coordinates)
predicted_morphology <- c()

for (coordinate in 1:dim(flattened_x_prima)[2]) {

  rlr_model <- rlm(flattened_x_prima[, coordinate] ~ as.matrix(pc_scores[-individual, ]))
  predicted_coordinate <- predict(rlr_model, as.data.frame(t(as.matrix(pc_scores[individual, ]))))[[1]]
  predicted_morphology <- c(predicted_morphology, predicted_coordinate)

}

predicted_morphology <- matrix(predicted_morphology, ncol = 3, byrow = TRUE)

#
```

Support Vector Machine

Because the SVM model does not produce multivariate outputs, the coordinates have to be predicted one by one. For this purpose we have to flatten the matrix, and then regress for each coordinate value separately.

```

flattened_x_prima <- vector_from_lanmdarks(x$coordinates)
predicted_morphology <- c()

for (coordinate in 1:dim(flattened_x_prima)[2]) {

  data_set <- as.data.frame(pc_scores[-individual, ])
  data_set$y <- flattened_x_prima[, coordinate]
  svm_model <- svm(y ~ ., data = data_set, kernel = "linear")
  predicted_coordinate <- predict(svm_model, as.data.frame(t(as.matrix(pc_scores[individual, ]))))[[1]]
  predicted_morphology <- c(predicted_morphology, predicted_coordinate)

}

predicted_morphology <- matrix(predicted_morphology, ncol = 3, byrow = TRUE)

#
```

Decision Tree

Because the DT model does not produce multivariate outputs, the coordinates have to be predicted one by one. For this purpose we have to flatten the matrix, and then regress for each coordinate value separately.

```r

flattened_x_prima <- vector_from_lanmdarks(x$coordinates)
predicted_morphology <- c()

for (coordinate in 1:dim(flattened_x_prima)[2]) {

  data_set <- as.data.frame(pc_scores[-individual, ])
  data_set$y <- flattened_x_prima[, coordinate]
  dt_model <- rpart(y ~ ., data = data_set, method = "anova", control = rpart.control(maxdepth = 3))
  predicted_coordinate <- predict(dt_model, as.data.frame(t(as.matrix(pc_scores[individual, ]))))[[1]]
  predicted_morphology <- c(predicted_morphology, predicted_coordinate)

}

predicted_morphology <- matrix(predicted_morphology, ncol = 3, byrow = TRUE)

#
```

Potentially Cleaner Implementation

In the above code we are controlling for Procrustes contamination, however a potential source of data leakage that is still potentially relevant is the fact that the predictors, z, are derived from potentially contaminated variables as well. The final version of the code implemented in the main study controlled for both sources of contamination - in the Procrustes coordiantes, and in the PC scores. We wish to argue, however, that there are fundamental differences in the framing of the training of a regression model and the definition of contamination in this sense. Including validation specimens in the GPA has a direct impact on the target space - the space we are trying to predict. Including the validation specimens in the PCA changes only the representation of the predictors. While it is ideal to control for both, it is not strictly necessary in real world examples. For the purpose of evaluating regression models, however, it is best to reduce contamination as much as possible to find the right model for the reconstruction task, and then the final reconstruction of the fossil can be performed as described above. For the purpose of evaluation, however, in this slightly cleaner implementation we redefine Algorithm 1 such that;

Algorithm 2: Alternative Leave-one-out evaluation workflow for landmark reconstruction

Input : Dataset (D) of n specimens
Output : Measurement Error

for $i \leftarrow 1$ **to** n **do**
- Split D into $D \setminus S_{val}$ (training) and S_{val} (validation), where $val = i$;
- Compute GPA without scaling on $D \setminus S_{val}$ to obtain the reference Procrustes coordinate system X;
- Remove the corresponding missing landmarks from $D \setminus S_{val}$ and perform GPA using only the training specimens to obtain the reduced landmark configuration X';
- Superimpose S_{val} onto this reduced landmark configuration using the Procrustes parameters obtained from the training specimens.;
- Compute morphological descriptors z from X' using PCA calculated only from the training specimens;
- Project S_{val} into this PCA space using the training PCA loadings;
- Establish a mapping $f : z \rightarrow X$;
- Use $f(z)$ to predict the coordinates of LM' for S_{val}, defining S'_{val};
- Append S_{val} to S'_{val} and X, and compute GPA without scaling;
- Compute RMSE between the superimposed coordinates of S_{val} and S'_{val};

```r
find_centroid <- function(configuration, robust = FALSE) {
  if (robust == FALSE) {
    centroid <- apply(configuration, 2, mean)
  } else {
    centroid <- apply(configuration, 2, median)
  }
  return(centroid)
}

centroid_size <- function(configuration, centroid) {
  distances <- c()
```

```
  for (lm in 1:nrow(configuration)) {
    distances <- c(distances, euclidean(configuration[lm,], centroid)^2)
  }
  return(sqrt(sum(distances)))
}

helmert <- function(p, type) {

  Helm_M <- matrix(0, p, p + 1)

  if (type == 1) {
    val <- 1
    while(val <= p) {
      val2 <- 1
      while(val2 <= val) {
        Helm_M[val, val2] <- -1 / sqrt(val * (val + 1))
        val2 <- val2 + 1
      }
      Helm_M[val, val + 1] <- val / sqrt(val * (val + 1))
      val <- val + 1
    }
  } else {
    if (p > 0) {
      for (val in seq(1, p)) {
        value = -1 / sqrt(val * (val + 1))
        Helm_M[val, seq(1, val)] = value
        Helm_M[val, val + 1] = - val * value
      }
    }
  }

  return(Helm_M)

}

kendall_preshape <- function(target_matrix, robust = FALSE) {

  p <- dim(target_matrix)[1]
  if (p < 100) {
    h_type = 1
  }  else {
    h_type = 2
  }
  h <- helmert(p - 1, h_type)

  preshape_x <- h %*% target_matrix
  centroid <- find_centroid(target_matrix,
                            robust = robust)
  CS <- centroid_size(target_matrix,
                      centroid)
  preshape_x <- preshape_x / CS

  return(preshape_x)

}

kendall_rotation <- function(matrix1, matrix2) {

  k <- dim(matrix1)[2]

  eigendecomp <- svd(t(matrix1) %*% matrix2)

  U_prima <- eigendecomp$v
  V <- eigendecomp$u
  Delta <- eigendecomp$d

  # remove reflection

  sign_det <- sign(det(t(matrix1) %*% matrix2))

```

```r
  Delta[k] <- sign_det * abs(Delta[k])
  V[,k] <- sign_det * V[,k]

  Gamma <- U_prima %*% t(V)

  return(Gamma)

}

rotate_shape <- function(shape, reference) {
  Gamma <- kendall_rotation(reference, shape)
  shape %*% Gamma
}

align_train_test_shapes <- function(train_array,
                                    validation_array,
                                    scale = TRUE,
                                    robust = FALSE) {

  train_aligned <- align_shapes(train_array, scale = scale, robust = robust)
  train_coords <- train_aligned$coordinates

  central_config <- if (robust) {
    calc_central_morph(train_coords, method = "median")
  } else {
    calc_central_morph(train_coords, method = "mean")
  }

  translate_shape <- function(shape, robust = FALSE) {
    centroid <- if (!robust) apply(shape, 2, mean) else apply(shape, 2, median)
    shape - matrix(centroid, nrow(shape), ncol(shape), byrow = TRUE)
  }

  p <- dim(train_coords)[1]
  k <- dim(train_coords)[2]
  n_train <- dim(train_coords)[3]
  Gamma_vec <- as.vector(kendall_preshape(central_config, robust = robust))
  Gamma_vec <- Gamma_vec / sqrt(sum(Gamma_vec^2))   # <- normalize to unit length
  identity_matrix <- diag(length(Gamma_vec))

  train_tangent <- matrix(0, length(Gamma_vec), n_train)
  for (i in 1:n_train) {
    train_tangent[,i] <- (identity_matrix - Gamma_vec %*% t(Gamma_vec)) %*%
      as.vector(kendall_preshape(train_coords[,,i], robust = robust))
  }

  p_test <- dim(validation_array)[1]
  k_test <- dim(validation_array)[2]
  n_test <- dim(validation_array)[3]
  test_aligned <- array(0, dim = c(p_test, k_test, n_test))

  for (i in 1:n_test) {
    shape <- validation_array[,,i]
    shape <- translate_shape(shape, robust = robust)
    if (scale) shape <- shape / centroid_size(shape, find_centroid(shape))
    shape <- rotate_shape(shape, central_config)
    test_aligned[,,i] <- shape
  }

  test_tangent <- matrix(0, length(Gamma_vec), n_test)
  for (i in 1:n_test) {
    test_tangent[,i] <- (identity_matrix - Gamma_vec %*% t(Gamma_vec)) %*%
      as.vector(kendall_preshape(test_aligned[,,i], robust = robust))
  }

  return(list(
    train_coords = train_coords,
    test_coords = test_aligned,
    train_tangent = train_tangent,
    test_tangent = test_tangent,
```

```
    central_config = central_config
  ))
}

n_PC <- 10
landmark_indices <- 26:38

residuals <- c()

for (individual in 1:dim(dataset$coords)[3]) {

    target <- array(
        dataset$coords[,,individual],
        dim = c(
            dim(dataset$coords)[1],
            dim(dataset$coords)[2],
            1
        )
    )

    training <- dataset$coords[,,-individual]

    full_alignment <- align_train_test_shapes(
        train_array = training,
        test_array = target,
        scale = FALSE
    )

    training_partial <- training[landmark_indices,,]

    target_partial <- target[landmark_indices,,]

    partial_alignment <- align_train_test_shapes(
        train_array = training_partial,
        test_array = target_partial,
        scale = FALSE
    )

    training_vectors <- t(
        vector_from_landmarks(
            partial_alignment$train_coords
        )
    )

    target_vector <- t(
        vector_from_landmarks(
            partial_alignment$test_coords
        )
    )

    pca_model <- prcomp(
        training_vectors,
        center = TRUE,
        scale. = FALSE
    )

    training_scores <- pca_model$x[,1:n_PC]

    target_scores <- predict(
        pca_model,
        newdata = target_vector
    )[,1:n_PC]

    predicted_morphology <- morphology_predictor(
        full_alignment$train_coords,
        training_scores,
        target_scores
    )

    aligned <- GPA(
```

```
        abind(
            dataset$coords[,,individual],
            predicted_morphology,
            along = 3
        ),
        scale = FALSE
    )

    original <- aligned$coordinates[,,1]

    reconstructed <- aligned$coordinates[,,2]

    landmark_errors <- c()

    for (landmark in 1:nrow(original)) {

        landmark_errors <- c(
            landmark_errors,
            euclidean(
                original[landmark,],
                reconstructed[landmark,]
            )
        )
    }

    residuals <- c(
        residuals,
        sqrt(mean(landmark_errors^2))
    )
}

#
```

Python Implementation

This section describes the Python implementation of a leave-one-out cross-validation (LOOCV) neural network used to predict the 3D coordinates of missing landmarks from principal component scores (PC scores). The workflow is designed to be general and applicable to any dataset of specimens with morphometric landmark coordinates. First we need to export the data from R so that it can be imported into Python for the training of the algorithm.

```

write.table(
  vector_from_landmarks(GPAform_all$coordinates),
  ".\\Form_Coordinates.txt",
  sep = ",",
  row.names = FALSE,
  col.names = FALSE
)

write.table(
  pc_scores_form,
  ".\\Form_PC_Scores.txt",
  sep = ",",
  row.names = FALSE,
  col.names = FALSE
)

#
```

The workflow then consists of the following steps:

1. Load the coordinate and PC score datasets for all specimens.
2. Define hyperparameters for the meta-learning loop, including:
   - **epsilon:** learning rate for meta-update
   - **num_tasks:** number of tasks per epoch
   - **num_samples:** number of samples per task
   - **mini_batch_size:** batch size for inner loop optimization
   - **epochs:** total number of epochs for meta-learning
   - **num_iterations:** inner loop optimization steps
   - **n_pc_scores:** number of PC scores used as input features
3. For each individual:
   (a) Load training coordinates (y) and PC scores (x) for all other specimens.
   (b) Load the PC scores for the test specimen (leave-one-out).
   (c) Construct a 3-layer fully-connected neural network with linear activations.
   (d) Perform a Reptile meta-learning loop:
      - Sample a mini-batch of training data.
      - Perform several gradient steps to update the model on the task.
      - Compute meta-update using epsilon-weighted difference between new and old parameters.

   (e) Save the final trained weights.
   (f) Predict the landmark coordinates for the test specimen using the trained network.
4. Save predictions for all individuals as CSV files for downstream analyses.

Dependencies

The following Python packages are required:

- **tensorflow** (v.2.12.0)
- **numpy** (v.1.23.5)
- **pandas** (v.2.3.3)
- **matplotlib** (v.3.9.2)

Please pay careful attention to the version of libraries used, as other versions of the libraries are likely to result in errors.

Metalearning Evalution Code

```

# set working directory to location of data files

import os
os.chdir("C:/Path/To/Your/Data/Folder")

# load standard packages

import numpy as np
import matplotlib
import matplotlib.pyplot as plt
import pandas as pd

# load Tensorflow

import tensorflow as tf
from tensorflow.keras.models import Sequential
from tensorflow.keras.layers import Dense

# disable eager execution for TF1 compatibility

import tensorflow.compat.v1 as tf1
tf1.disable_eager_execution()

# Check library requirements

print("Python versions -------------")
print(f"Numpy:  {np.__version__}")
print(f"Matplotlib:  {matplotlib.__version__}")
print(f"TensorFlow:  {tf.__version__}")
print(f"Pandas:  {pd.__version__}")

# set hyperparameters for meta-learning

number_of_individuals = int(len(os.listdir()) / 2)
epsilon = 0.1
num_tasks = 10
num_samples = 30
mini_batch_size = 10
epochs = 200
num_iterations = 10
n_pc_scores = 10

# loop over individuals for leave-one-out cross-validation

os.makedirs("Reptile Files", exist_ok = TRUE)

for i in range(1, number_of_individuals + 1):

    x_name = f"Individual_{i}_train_x_for_NN.txt"
    z_name = f"Individual_{i}_train_z_for_NN.txt"
    individual_test = f"Individual_{i}_test_z_for_NN.txt"

    y = np.loadtxt(x_name, delimiter = ",")  # load coordinates
```

```
    x = np.loadtxt(z_name, delimiter = ",")  # load PC scores
    target_pc = np.loadtxt(individual_test, delimiter = ",")

    input_size = int(x.shape[1])
    hidden_layer_size = int(np.ceil(x.shape[1] + (x.shape[1] * 0.5)))
    output_size = int(y.shape[1])

    tf1.reset_default_graph()

    # define placeholders

    x_place = tf1.placeholder(tf.float32, shape = [None, input_size])
    y_place = tf1.placeholder(tf.float32, shape = [None, output_size])

    # initialize weights and biases

    theta_1 = tf1.Variable(tf1.random_uniform([input_size, hidden_layer_size]))
    bias_1 = tf1.Variable(tf1.random_uniform([hidden_layer_size]))

    theta_2 = tf1.Variable(tf1.random_uniform([hidden_layer_size, hidden_layer_size]))
    bias_2 = tf1.Variable(tf1.random_uniform([hidden_layer_size]))

    theta_3 = tf1.Variable(tf1.random_uniform([hidden_layer_size, output_size]))
    bias_3 = tf1.Variable(tf1.random_uniform([output_size]))

    # define linear forward pass

    z1 = tf.matmul(x_place, theta_1) + bias_1
    a1 = z1

    z2 = tf.matmul(a1, theta_2) + bias_2
    a2 = z2

    z3 = tf.matmul(a2, theta_3) + bias_3
    yhat = z3

    loss_function = tf1.reduce_mean(tf.square(yhat - y_place))
    optimizer = tf1.train.AdamOptimizer(1e-2).minimize(loss_function)
    init = tf1.global_variables_initializer()

    training_loss = np.array([])

    # start Tensorflow session for training

    with tf1.Session() as sess:

        sess.run(init)

        for epoch in range(epochs):

            for task in range(num_tasks):

                # save old parameters

                old_theta_1, old_bias_1, old_theta_2, old_bias_2, old_theta_3, old_bias_3 = sess.run(
                    [theta_1, bias_1, theta_2, bias_2, theta_3, bias_3]
                )

                # sample mini-batch for task

                sample_index = np.random.randint(x.shape[0], size = num_samples)
                x_train = x[sample_index,]
                y_train = y[sample_index,]

                # inner loop optimization

                for k in range(num_iterations):

                    for minibatch in range(0, num_samples, mini_batch_size):

```

```
125                         x_minibatch = x_train[minibatch:minibatch + mini_batch_size]
126                         y_minibatch = y_train[minibatch:minibatch + mini_batch_size]
127
128                         _ = sess.run(
129                             optimizer,
130                             feed_dict = {x_place: x_minibatch, y_place: y_minibatch}
131                         )
132
133                 # meta-update
134
135                 new_theta_1, new_bias_1, new_theta_2, new_bias_2, new_theta_3, new_bias_3 = sess.run(
136                     [theta_1, bias_1, theta_2, bias_2, theta_3, bias_3]
137                 )
138
139                 theta_1.load(old_theta_1 + epsilon * (new_theta_1 - old_theta_1), sess)
140                 bias_1.load(old_bias_1 + epsilon * (new_bias_1 - old_bias_1), sess)
141                 theta_2.load(old_theta_2 + epsilon * (new_theta_2 - old_theta_2), sess)
142                 bias_2.load(old_bias_2 + epsilon * (new_bias_2 - old_bias_2), sess)
143                 theta_3.load(old_theta_3 + epsilon * (new_theta_3 - old_theta_3), sess)
144                 bias_3.load(old_bias_3 + epsilon * (new_bias_3 - old_bias_3), sess)
145
146             loss = sess.run(loss_function, feed_dict = {x_place: x_train, y_place: y_train})
147             training_loss = np.append(training_loss, loss)
148
149         # extract final trained parameters
150
151         theta_1 = theta_1.eval()
152         bias_1 = bias_1.eval()
153         theta_2 = theta_2.eval()
154         bias_2 = bias_2.eval()
155         theta_3 = theta_3.eval()
156         bias_3 = bias_3.eval()
157
158     # create Keras model with learned weights
159
160     meta_nn_regressor = Sequential()
161     meta_nn_regressor.add(Dense(hidden_layer_size, input_shape = (n_pc_scores,)))
162     meta_nn_regressor.add(Dense(hidden_layer_size))
163     meta_nn_regressor.add(Dense(y.shape[1]))
164     meta_nn_regressor.layers[0].set_weights([theta_1, bias_1])
165     meta_nn_regressor.layers[1].set_weights([theta_2, bias_2])
166     meta_nn_regressor.layers[2].set_weights([theta_3, bias_3])
167
168     # make leave-one-out prediction
169
170     loo_prediction = meta_nn_regressor.predict(target_pc.reshape(1, -1))
171
172     np.savetxt(
173         f"./Reptile Files/Meta_NN_Pred_Individual_{i}.txt",
174         loo_prediction.reshape(200,3),
175         delimiter = ","
176     )
177     print(f"Individual {i} done")
178
179     #
```

The core of the metalearning regression algorithm is therefore from lines 58 to 166, while line 170 is used to perform new predictions.

Data can then be studied in R in a similar fashion to what appears in section 9, if the txt files are loaded into R the residuals can then be evaluated.

```r

# Evaluate Error

residuals <- c()

# Loop over all individuals

number_of_individuals <- length(
    list.files(
        ".\\Reptile Files",
        pattern = "Meta_NN_Pred_Individual_.*\\.txt"
    )
)

for (i in 1:number_of_individuals) {

  # Load the predicted coordinates for individual i

  predicted_file <- paste0(
      ".\\Reptile Files\\Meta_NN_Pred_Individual_", i, ".txt"
  )
  reconstructed_coordinates <- as.matrix(read.table(predicted_file, sep = ",", header = FALSE))

  # Get the original coordinates for this individual

  original_coordinates <- GPAform_all$coordinates[(1:200)[-landmark_indices], , i]

  # Compute residuals per landmark

  landmark_residual <- numeric(nrow(reconstructed_coordinates))

  for (landmark in 1:nrow(reconstructed_coordinates)) {

    landmark_residual[landmark] <- euclidean(
      reconstructed_coordinates[landmark, ],
      original_coordinates[landmark, ]
    )

  }

  residuals[i] <- rmse(landmark_residual)

}

# Summarize residuals across all individuals

describe_residuals(residuals)

#
```

Potentially Cleaner Implementation

Note that in order for the python metalearning implementation to be done as proposed by Algorithm 2 then it is best that the landmark coordinates be exported from R before the PCA, and the PCA be conducted per specimen directly in python to define z. PCA in python can be done using the Scikit-learn library.
